# Conservation and global distribution of non-canonical antigens in Enterotoxigenic *Escherichia coli*

**DOI:** 10.1371/journal.pntd.0007825

**Published:** 2019-11-22

**Authors:** F. Matthew Kuhlmann, John Martin, Tracy H. Hazen, Tim J. Vickers, Madeline Pashos, Pablo C. Okhuysen, Oscar G. Gómez-Duarte, Elizabeth Cebelinski, Dave Boxrud, Felipe del Canto, Roberto Vidal, Firdausi Qadri, Makedonka Mitreva, David A. Rasko, James M. Fleckenstein

**Affiliations:** 1 Department of Medicine, Division of Infectious Diseases, Washington University School of Medicine, St. Louis, Missouri, United States of America; 2 McDonnell Genome Institute, Washington University School of Medicine, St. Louis, Missouri, United States of America; 3 Department of Microbiology and Immunology and Institute for Genome Sciences, University of Maryland School of Medicine, Baltimore, Maryland, United States of America; 4 The Department of Infectious Diseases, Infection Control and Employee Health, University of Texas M.D. Anderson Cancer Center, Houston, Texas, United States of America; 5 Department of Pediatrics, Division of Infectious Diseases, University at Buffalo, The State University of New York, Buffalo, New York, United States of America; 6 Minnesota Department of Health, St. Paul, Minnesota, United States of America; 7 Programa de Microbiología y Micología, Instituto de Ciencias Biomédicas, Facultad de Medicina, Universidad de Chile, Santiago, Chile; 8 Instituto Milenio de Inmunonología e Inmunoterapia, Facultad de Medicina, Universidad de Chile, Santiago, Chile; 9 Infectious Diseases Division, International Centre for Diarrhoeal Disease Research, Bangladesh (icddr,b), Dhaka, Bangladesh; 10 Medicine Service, Veterans Affairs Medical Center, St. Louis, Missouri, United States of America; University of Texas Medical Branch, UNITED STATES

## Abstract

**Background:**

Enterotoxigenic *Escherichia coli* (ETEC) cause significant diarrheal morbidity and mortality in children of resource-limited regions, warranting development of effective vaccine strategies. Genetic diversity of the ETEC pathovar has impeded development of broadly protective vaccines centered on the classical canonical antigens, the colonization factors and heat-labile toxin. Two non-canonical ETEC antigens, the EtpA adhesin, and the EatA mucinase are immunogenic in humans and protective in animal models. To foster rational vaccine design that complements existing strategies, we examined the distribution and molecular conservation of these antigens in a diverse population of ETEC isolates.

**Methods:**

Geographically diverse ETEC isolates (n = 1159) were interrogated by PCR, immunoblotting, and/or whole genome sequencing (n = 46) to examine antigen conservation. The most divergent proteins were purified and their core functions assessed *in vitro*.

**Results:**

EatA and EtpA or their coding sequences were present in 57.0% and 51.5% of the ETEC isolates overall, respectively; and were globally dispersed without significant regional differences in antigen distribution. These antigens also exhibited >93% amino acid sequence identity with even the most divergent proteins retaining the core adhesin and mucinase activity assigned to the prototype molecules.

**Conclusions:**

EtpA and EatA are well-conserved molecules in the ETEC pathovar, suggesting that they serve important roles in virulence and that they could be exploited for rational vaccine design.

## Author summary

Enterotoxigenic *Escherichia coli* (ETEC) are a significant cause of childhood diarrhea in low-middle income countries for which there is currently no vaccine. ETEC vaccine approaches to date have focused on antigens known as colonization factors (CFs) and the pathovar-defining toxins. However, both antigenic diversity of CFs and the lack of characterized CF antigens in many strains has hindered deployment of broadly protective ETEC vaccines. Nevertheless, more recent studies have identified additional ETEC virulence molecules that could compliment canonical vaccine strategies. Here, we demonstrate that two proteins, the EtpA adhesin and EatA mucinase, are present in a diverse, global collection of isolates, and are among the most highly conserved ETEC pathovar-specific antigens to be identified, providing additional incentive to explore their utility in the rational design of broadly protective ETEC vaccines.

## Introduction

Enterotoxigenic *Escherichia coli* (ETEC) are a genetically heterogeneous group of pathogens defined by the production of plasmid-encoded heat-labile (LT) and/or heat-stable (ST) enterotoxins [1]. Diarrheal illness caused by ETEC ranges from mild to severe cholera-like disease [2]. ETEC is a significant cause of severe diarrhea in young children of low-middle income countries [3, 4], where it leads to tens of thousands of deaths each year. The burden of illness expands substantially with hundreds of millions of less severe cases of diarrhea attributable to ETEC [5]. Childhood infection with ETEC [5–8] is associated with substantial morbidity and post-diarrheal sequelae including environmental enteropathy, malnutrition, growth stunting, and cognitive impairment [9–12]. The strong association of malnutrition and deaths due to other infectious diseases further amplifies the potential impact of an effective vaccine [4, 12, 13].

Given that a large proportion of the world population remains without ready access to clean water or basic sanitation, vaccines targeting ETEC and other common enteric pathogens, remain a high priority in efforts to prevent childhood diarrheal illness [14]. The canonical approach to ETEC vaccine development is based on a classical virulence paradigm in which the bacteria adhere to the small intestine via plasmid-encoded colonization factors (CFs) [15] allowing delivery of their ST and/or LT enterotoxin payloads causing secretory diarrhea. Although the relationship of anti-CF antibodies to protection following ETEC infections in endemic areas has been difficult to establish [16], controlled human infection studies convincingly demonstrate that passive immunization against CFs, or CF tip adhesin molecules can prevent symptomatic ETEC diarrhea [17]. Although most ETEC appear to encode CFs or CF-like chaperone-usher-pilus operons [18] 20–50% of isolates lack any of the 25 established and immunologically distinct CFs that have been characterized to date [19, 20], potentially confounding development of a broadly protective vaccine focused exclusively on CFs, and prompting the exploration of additional antigens, including toxoids that could collectively afford broad-based protection [21–23].

Human challenge studies demonstrated that the ETEC H10407 strain cured of a virulence plasmid encoding CFA/I was avirulent, engendering early enthusiasm for targeting CFs [24]. Notably, subsequent genetic and molecular pathogenesis studies identified two additional pathovar-specific, non-canonical virulence loci on this plasmid; *eatA*, which encodes a member of the serine protease autotransporters of *Enterobacteriaceae* (SPATE) family [25], and the *etpBAC* two-partner secretion adhesin locus [26]. EatA has been shown to facilitate interaction with epithelial cells by limiting accumulation of the secreted EtpA adhesin [27] and by degrading MUC2, the major mucin secreted by goblet cells in the intestinal mucosa [28]. EtpA is a large (170kD) extracellular glycoprotein that forms a molecular bridge from the bacterial surface to intestinal epithelia where it interacts with abundant N-acetylgalactosamine (GalNAc) residues of mucosal glycoproteins [29]. Recently, EtpA was shown to interact preferentially with GalNAc residues presented as the terminal glycan on the A blood group, thereby accelerating bacterial adhesion and toxin delivery. This interaction may underlie the enhanced disease severity following ETEC infections in blood group A hosts [6, 30].

To date, studies of EtpA and EatA suggest that these non-canonical antigens could complement classical approaches to ETEC vaccine development. Each of the proteins have been shown to protect against ETEC infection in a murine model of intestinal colonization [28, 31] and are immunologically recognized following experimental human challenge [32] or natural infection with ETEC [33].

Because underlying plasticity of *E*. *coli* genomes can complicate vaccine development [34], antigenic conservation across a broad representation of isolates is of paramount importance in ETEC antigen selection. Although an earlier study, focused primarily on isolates from Bangladesh, suggested that both the *eatA* and *etpBAC* loci were present in a phylogenetically distributed collection of ETEC isolates [33], further studies are required to assess the global distribution of each locus and the functional conservation of the respective proteins within the genetically diverse ETEC pathovar.

In the present study, we examined the molecular and functional conservation of EatA and EtpA in a large, unbiased collection of validated ETEC strains isolated from cases of symptomatic diarrhea in the Global Enteric Multicenter Study (GEMS) [4] complemented with isolates from regions of the Western Hemisphere not represented in GEMS. The interrogation of this global collection of isolates demonstrates that EtpA and EatA are among the most highly conserved ETEC pathovar-specific virulence molecules discovered to date, findings that should foster further exploration of their role in disease and as protective antigens.

## Methods

### Ethics statement

All bacterial samples analyzed in these studies were de-identified and obtained from previously archived existing collections outlined in S5 Table.

### Sample acquisition

To obtain a diverse and unbiased collection, well-validated ETEC isolates were obtained from a variety of sources. These included 883 ETEC isolates from children with moderate-severe diarrhea in the Global Enteric Multicenter Study (GEMS) obtained through the University of Maryland [4]. Toxin characterization for these isolates was performed at the study site as previously described [35] and verified using PCR with primers described in S1 Table. CF profiles were determined by Roberto Vidal at Universidad de Chile using polymerase chain reaction as described elsewhere [20]. Eight ETEC isolates were obtained from U.S. and Canadian adults with traveler’s diarrhea while visiting Mexico [36], and an additional 40 isolates were provided by the Minnesota Department of Health [37]. Those from Bangladesh (icddr,b) included 171 previously collected isolates [33] and 50 additional isolates collected in the course of routine 2% molecular surveillance studies of acute watery diarrhea conducted at icddr,b. Additional clinical isolates from Chile [38], Colombia [39, 40], and elsewhere had also been previously characterized by PCR and/or western blotting (S2 Table).

### Antigen detection by PCR and immunoblotting

ETEC were grown overnight in 3 ml Luria Broth at 37°C from frozen glycerol stock stored at -80°C. Total nucleic acids were extracted using the Wizard Genomic DNA Purification Kit (Promega catalog #A1120) according to the provided protocol and resuspended in 200 μl water. PCR was performed using primers in S1 Table and described in supporting methods. Supernatants from the overnight cultures used for nucleic acid extraction underwent trichloroacetic acid precipitation and immunoblotting as previously described [33]. Full details of the immunoblotting protocol are available in supporting methods.

### Adjudication of discordant results

Samples with discordant PCR/immunoblot data underwent repeat culture from the same stock and DNA was extracted using phenol/chloroform extraction and ethanol precipitation (supporting methods). Immunoblotting was also performed on the repeat culture. A third assay on a separate culture was performed if repeat testing did not confirm our prior results. If the third assay again gave differing or ambivalent results, such as non-specific PCR products, the isolate was not included in our analysis (S1 Fig). EatA positive samples with negative PCR and positive western blots were included as positive in our final analysis, as negative PCR data may result from minor sequence divergence at the primer binding site(s).

### Genome sequencing

A total of 46 EtpA positive strains representing diverse geographic origins, CFs, and toxin profiles were selected for Illumina whole genome sequencing (S3 Table). Phenol/chloroform extraction was performed on pelleted cells from 3 ml overnight cultures; *etpA* presence was confirmed by PCR prior to whole genome sequencing. Automated dual indexed libraries were constructed with 600ng of genomic DNA utilizing the KAPA Hyper PCR-free Library Kit (KAPA Biosystems) on the SciClone NGS instrument (Perkin Elmer) targeting 350bp inserts. The concentration of each library was accurately determined through qPCR (Kapa Biosystems) in order to make 250pM library dilutions for the HiSeqX platform. 2x151bp paired end sequence data generated approximately 2.2 Gb per sample. Sequencing runs were completed according the manufacturer’s recommendations (Illumina Inc, San Diego, CA). A subset of strains (S3 Table) underwent individual strain or pooled, indexed PacBio sequencing. SMRTbell libraries were prepared starting with 4ug material per each of 12 samples. Samples were mechanically sheared using the Covaris AFA system and then size selected for 10kb fragments using PacBio’s BluePippin system. 500ng per sample of fragmented material (as quantified by Qubit fluorometer) were used to build a single, pooled library. This barcoded library was sequenced on a single SMRT cell using PacBio Sequel 2.0 chemistry. Individual libraries were de-multiplexed from the pool after sequencing using PacBio’s SMRT Link. The H10407 and Jurua 18/11 samples followed the same method through shearing, but then 1ug of 10kb fragmented material was used to generate 2 additional libraries that were sequenced on the PacBio RSII using P6-C4 chemistry and assembled using HGAP4 default settings within the SMRT assembler. A hybrid assembly of the 12 indexed samples was performed using the SPAdes assembler. Trimmomatic cleaned, paired end Illumina data and PacBio Sequel subread data for each sample were fed to the SPAdes assembler using default assembly parameters. Job parameters included '--threads 4' and '--memory 29', recruiting 4 cpus and 29Gb of memory per hybrid assembly. These hybrid assemblies proved more contiguous than earlier HGAP 4 assemblies (using default HGAP4 arguments) over the *etpBAC* locus.

### Sequence alignment and phylogenetic trees

Whole genome sequence was trimmed using Trimmomatic version 0.36 [41], then mapped to the H10407 p948 plasmid as a reference (GenBank: FN649418.1) using HISAT2 version 2.1.0 (https://ccb.jhu.edu/software/hisat2/index.shtml) using default settings. Consensus sequence was obtained for the H10407 *etpA* gene (GenBank: NC_017724) using the integrated genomics viewer (http://software.broadinstitute.org/software/igv/) default settings [42]. Raw reads from previously published isolates were downloaded from the GenBank’s SRA and were mapped to the same reference plasmid (Assembly: ASM21047v1) to confirm the veracity of our method (S3 Table, Bioproject PRJNA526881). Whole genome consensus sequence was uploaded to European Bioinformatics Institute and phylogenetic trees were generated using Clustal Omega (https://www.ebi.ac.uk/Tools/msa/clustalo/) and visualized using FigTree version 1.4.3 (http://tree.bio.ed.ac.uk/software/figtree/).

### Recombinant protein purification

The most divergent strains were selected based on their observed distance in assembled phylogenetic trees described above. jf4927 and jf4894 (S4 Table) expression strains were grown overnight in 100 ml Luria Broth with 100 μg/ml ampicillin and 15 μg/ml chloramphenicol then diluted to 4 L until reaching an OD 600 of 0.6. Expression was induced with 0.0002% (w/v) arabinose for 6 hours at 37°C. After centrifugation, culture supernatants were concentrated using 100kD Molecular Weight Cutoff tangential-flow filter (Millipore). The secreted his-tagged protein was purified using immobilized metal affinity chromatography as described [30, 43]. Strain jf5003 expressing EatA from strain 700241 was utilized for recombinant protein expression and purification as previously described with the omission of ammonium sulfate precipitation [28].

### Functional assays

EtpA binding to blood group A-expressing HT-29 cells and the CRISPR α1-3-*N*-acetylgalactosaminyltransferase deletion line (IE6), generating an isogenic blood group O line [44], was assessed using immunofluorescence (supporting methods) and fluorescence signal was quantified using Volocity software (v6.3).

The rate of recombinant EatA (30 μg) cleavage of N-Succinyl-Ala-Ala-Pro-Leu p-nitroanilide, a common substrate for serine proteases, was performed as previously described without the inclusion of ZnCl_2_ [25] while MUC2 cleavage was performed as described [28] (supporting methods).

### Statistics

A master database of metadata was maintained in Excel 2010 and imported into SPSS (v24) for analysis including determinations of means and 95% confidence intervals (S2 Table). Chi-square analysis was used to determine differences in *etpA*/EtpA or *eatA*/EatA distribution between geography, study origin, and each colonization factor or toxin. Under or over-representation for plasmid associated genes was determined using binomial distributions in Excel (2016) and adjusted for multiple comparisons with a value of <0.05 determining significance (https://www.sdmproject.com/utilities/?show=FDR).

## Results

### Global conservation of EatA and EtpA

Rational vaccine design necessitates a thorough understanding of antigenic conservation throughout a diverse population. Given the extraordinary genetic plasticity of *E*. *coli* [45], the diversity of canonical ETEC target antigens [19, 20], and the worldwide distribution of ETEC, we sought an unbiased and global approach to comprehensively determine the molecular distribution of these candidate antigens. Utilizing isolates collected from the GEMS study [4] which included 7 sites in Africa and Asia, complemented with additional isolates obtained from patients in Bangladesh, Chile [38], Colombia [39, 40], the Minnesota Department of Health [37], and Mexico [36], we employed genetic and immunologic detection methods to determine the distribution of both antigens (S2 Table). There was strong concordance between the identification of either *etpA* or *eatA* genes by PCR with production of the corresponding protein determined by immunoblotting (r = 0.82 for *eatA*/EatA and 0.92 for *etpA*/EtpA). Overall, these antigens were identified in at least half of all isolates with the *eatA* gene and/or EatA protein present in 57.0% (95% CI 54.2–59.9%) and *etpA* gene and/or EtpA protein detected in 51.5% (48.6–54.4%). Combined, 73.3% (70.7–75.8%) of all isolates encode either EatA or EtpA (Table 1).

**Table 1:** Distribution of coding regions and/or protein expression for EatA and EtpA.

Region	Total Isolates	*eatA* or EatA			*etpA* or EtpA			*eatA*/EatA or *etpA*/EtpA		
		N	%	95%CI	N	%	95%CI	N	%	95%CI
Asia	524	301	57.4	53.2–61.7	278	53.1	48.8–57.3	391	74.6	70.9–78.4
Africa	516	297	57.6	53.3–61.8	258	50.0	45.7–54.3	378	73.3	69.4–77.1
North, Central, and South America	105	55	52.4	42.7–62.1	54	51.4	41.7–61.2	70	66.7	57.5–75.8
Europe or Unknown	14	8	57.1	27.5–86.8	7	50.0	20.0–80.0	10	71.4	44.4–98.5
Total	1159	661	57	54.2–59.9	597	51.5	48.6–54.4	849	73.3	70.7–75.8

*N = Number of positive isolates for specified condition

Geographical variation in ETEC antigen conservation may occur for multiple reasons including founder effects, clonality due to localized epidemics, or sampling within a limited timeframe. However, we found no appreciable difference in the occurrence of either antigen according to geographical distribution (Table 1, *eatA*/EatA *p* = 0.80, *etpA*/EtpA *p* = 0.81, either antigen *p* = 0.42, Chi-squared testing). Similarly, variation between studies could also arise for technical reasons such as differences in isolation procedures, culture or storage techniques, and temporal trends during sample collection. Nevertheless, we found no differences in the distribution of these antigens based on the collection (S5 Table, Chi-square testing for *eatA*/EatA *p* = 0.706, *etpA*/EtpA *p* = 0.214, or either *p* = 0.220).

To account for potential plasmid loss that could impact determination of antigen distribution, we verified the presence of genes encoding ETEC-defining toxins (S1 Fig). Eighty-four of the original 1243 isolates (6.8%) were determined not to be ETEC or to have lost their toxin-encoding plasmids, consistent with prior estimates of the frequency of plasmid loss due to culture passage alone [16, 46]. The majority of excluded strains were from the GEMS dataset (77 of 84, 91.7%).

### EatA and EtpA conservation relative to colonization factors and toxins

**Fig 1:**
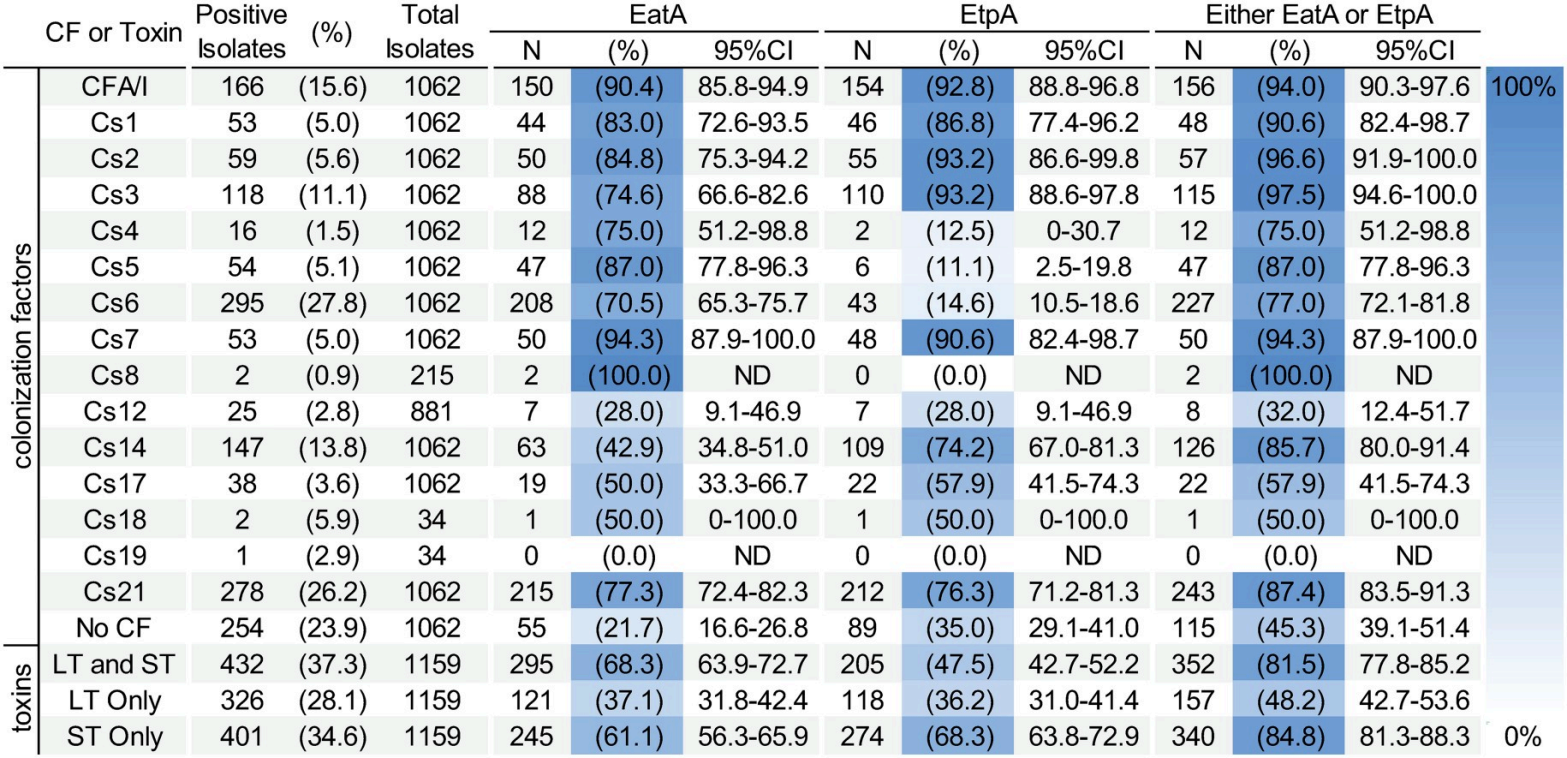
EatA or EtpA co-existence within ETEC strains expressing unique colonization factors and toxin types. Number for column headings represents the total number positive for the specified condition. Isolates expressing both CFs and the designated non-canonical antigen are depicted.

**Fig 2:**
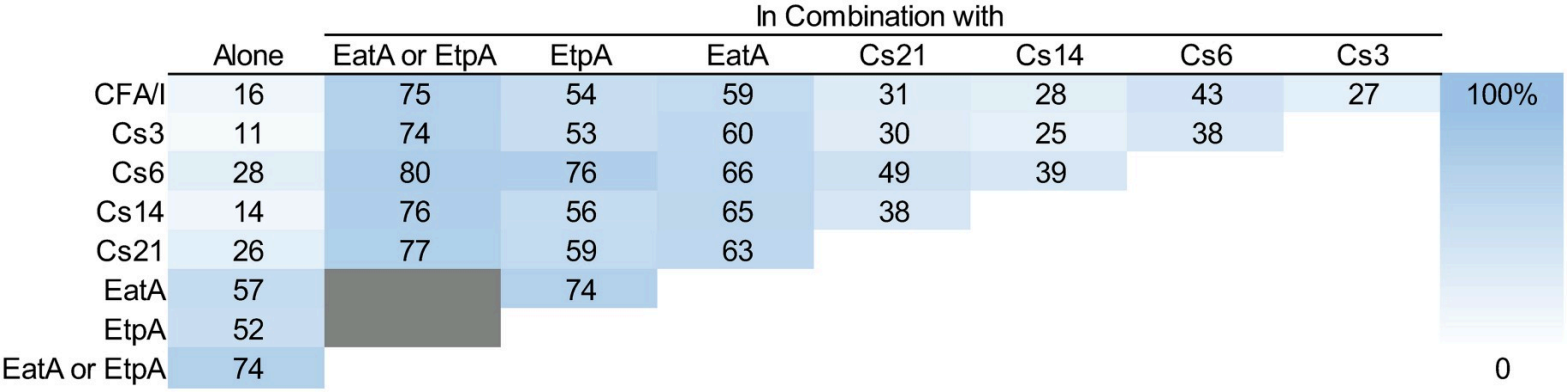
Percentage of ETEC strains expressing unique antigens alone or in combination with each other. The percentage of ETEC strains expressing either antigen is presented. Only CFs found in ≥ 10% of the collection are included. Antigen combinations were determined for isolates expressing either antigen.

EatA or EtpA could augment current vaccine strategies by expanding antigenic valency and by targeting different virulence mechanisms [47, 48]. To further assess the utility of EatA and EtpA as candidate antigens, we determined their conservation relative to each of the major ETEC colonization factors. In general, we found that either EtpA or EatA were conserved among isolates which expressed major colonization factors (CFA/I, CS1, CS2, CS3, and CS7). However, similar to earlier surveys conducted in Bangladesh [33], EtpA was underrepresented in strains that express CS4, CS5, and CS6, Fig 1). Encouragingly, while 23.9% of strains lacked an identifiable CF, *eatA*/EatA or *etpA*/EtpA were found in nearly half of these isolates (115 of 254, 45.3%), suggesting that these antigens could complement canonical approaches by expanding antigenic coverage. We also examined the distribution of EatA or EtpA in combination with individual CFs having a baseline occurrence of >10% (Fig 2). As predicted, given the underrepresentation of EtpA with Cs6 expressing isolates, the greatest increases are observed when combining CS6 with EtpA. Similar to the canonical CFs [20], we also found that the *eatA* and *etpBAC* loci were more commonly found in ST and ST/LT strains than those producing only LT.

### EtpA sequence and functional conservation

The large and globally diverse strain collection affords the opportunity to identify sequence variation that may alter either the function or immunogenic epitopes of EatA and EtpA. We performed whole genome sequencing (Illumina) of 46 EtpA-expressing strains of which 29 co-expressed EatA and supplemented our collection with sequence available in GenBank (S3 Table). The selected strains represent diverse geographies, toxin profiles, and CFs in an attempt to maximize potential sequence heterogeneity.

**Fig 3:**
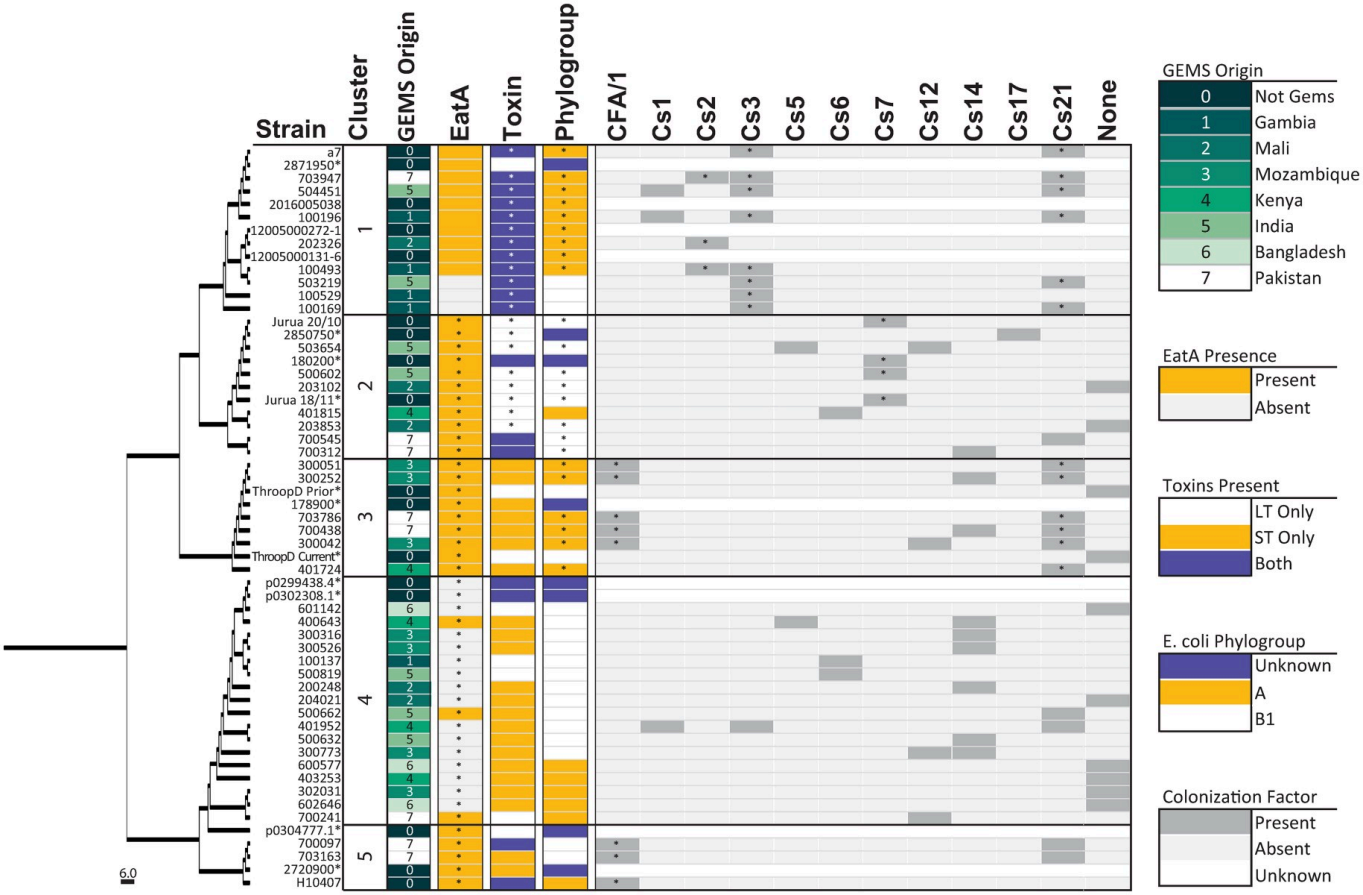
Phylogenetic distribution of EtpA sequences and associated metadata. Five clusters of EtpA were identified using Clustal Omega and FigTree software. Isolates marked with an asterisk denote sequences downloaded from the NCBI as external references (S3 Table). Within boxes, asterisks represent significant under (*e*.*g*., EatA absence in cluster 4) or over representation of the specified variable between clusters as calculated using binomial distributions for each variable. For CFs, “None” designates no CF was identified by PCR. Unknown means we have no data to support the presence or absence of a CF or were not included in the phylogroup analysis. Significant *p*-values were adjusted for multiple comparisons (* = *p* < 0.05).

Notably, the overall EtpA sequence identity was high (93.6–100%, N = 56) and comparable to sequence diversity of *eltAB* genes, encoding the heat-labile toxin of ETEC [20, 49]. Likewise, when the minor sequence variations in *etpA* sequences were mapped onto the prototype sequence of the molecule from H10407 (Accession NC_017724) and used to generate different phylogenic clusters, there was no clear association of any particular sequence cluster with geographic location (Fig 3). Using whole genome phylogrouping [50], each of the sequenced genomes mapped to phylogroups A and B1 (S2 Fig), where we observed unexpected yet significant overrepresentation of *E*. *coli* phylogroups within EtpA clusters, suggesting certain phylogroups are permissive to or co-evolved with distinct ETEC plasmids.

Short-read sequencing of *etpA* can be confounded by multiple, large repetitive sequences located at the 3’ region of the gene spanning 3.34 kb. Therefore, to confirm the sequence of the *etpA* repeat region, we performed additional long read sequencing (PacBio) of *etpA* from ETEC strains H10407, Jurua 18/11, and 100169. The resulting *de novo* assemblies were then compared to sequence assemblies generated using short-read (Illumina) technology. H10407 and Jurua 18/11 were individually sequenced by PacBio and assembled using HGAP4 default settings with mean read length of 10.8 kb for H10407 and 12.0 kb for Jurua 18/11. Indexed PacBio sequencing of 100169 yielded sub-reads of 3.8 kb as described in materials and methods. Very few differences were observed between the sequences generated by PacBio vs Illumina methodologies with (96.7% of bases identical for 1001696, 98.7 for Jurua 18/11, and 100% for H10407). Collectively, these data suggest that EtpA exhibits very little sequence variation over its geographic distribution.

**Fig 4:**
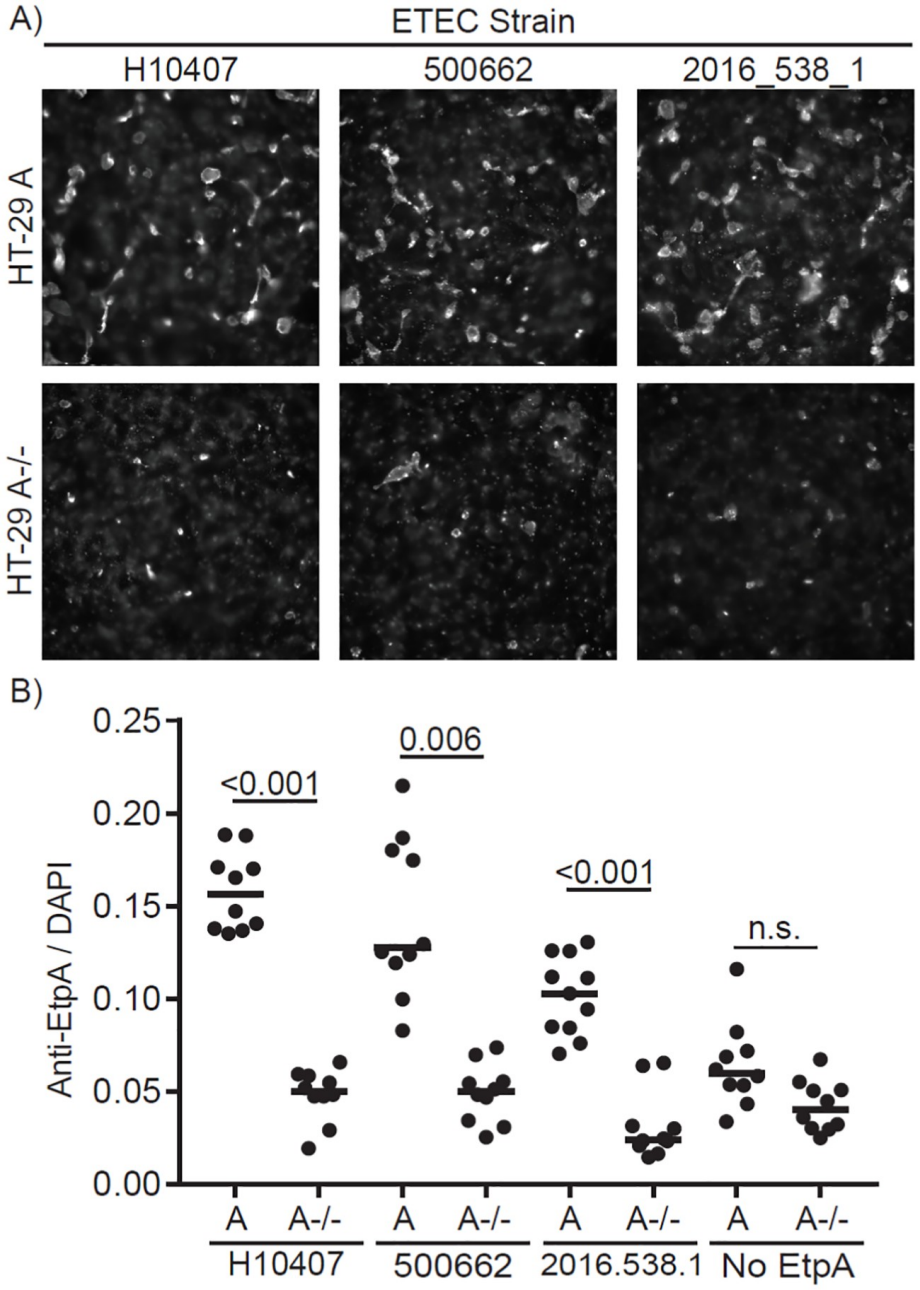
EtpA function is conserved. (A) Immunofluorescence microscopy of EtpA bound to HT-29 WT cells expressing Blood Group A sugars or HT29 A-/- CRISPR deletion mutant generating functional blood group O cells, EtpA detected with anti-EtpA antibodies. (B) Quantitation of mean fluorescent values normalized to DAPI (nuclei) signal using Volocity software. Statistical differences determined by Kruskal-Wallis testing followed by Dunn’s test for multiple comparisons with *p*<0.05 considered significant.

EtpA from H10407 binds preferentially to N-acetylgalactosamine expressed in the context of A blood group glycans on the intestinal mucosa, likely accounting for the increased disease severity observed in individuals with this blood group [6, 30]. To assess functional conservation of the protein, we cloned the most divergent *etpA* genes, expressed and purified the corresponding recombinant EtpA proteins, and examined their interaction with blood group A glycans. Similar to the H10407 prototype molecule, EtpA from these divergent strains exhibited preferential binding to blood group A carbohydrates expressed on the surface of enterocytes (Fig 4), suggesting functional conservation across a broad representation of ETEC.

### EatA sequence and functional conservation

**Fig 5:**
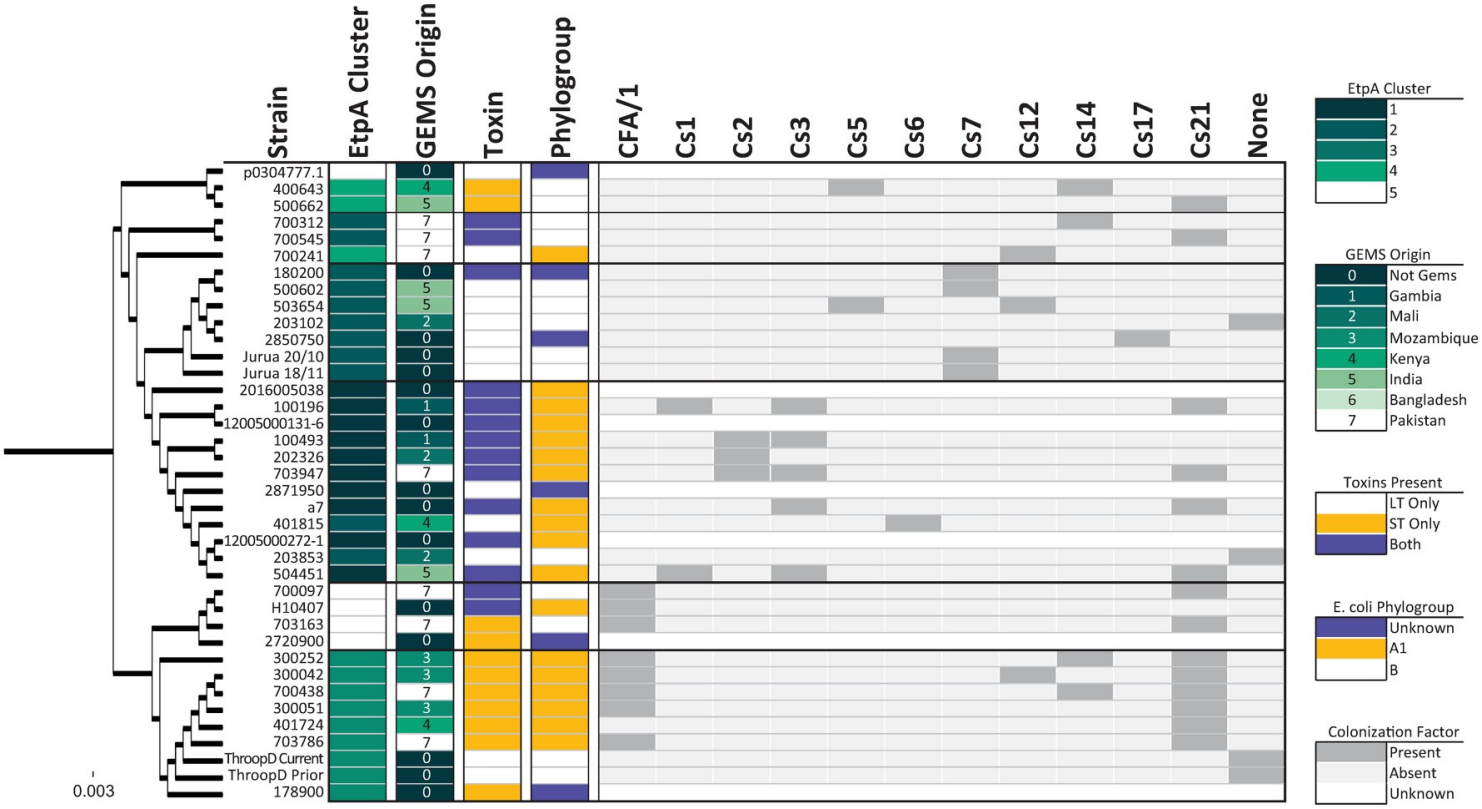
Phylogenetic distribution of EtpA sequences and associated metadata. Phylogenetic differences between EatA isolates and associated metadata using the same methods as in Fig 1. No statistics were determined for this data set due to small numbers and inherent bias in the data.

We also assessed potential sequence and functional differences in EatA expressing isolates. The percent amino acid identities for EatA ranged from 94.3 to 100% (N = 38) relative to the H10407 reference sequence. The EatA sequences cluster in a similar fashion to EtpA, a finding we expect as both genes are encoded on the same plasmid in H10407 (Fig 5). Importantly, evaluation of the predicted EatA protein sequence demonstrated complete conservation of the catalytic residues comprised of H134, D162, and S267 (S3 Fig). Similarly, comparison of five available *eatA* sequences from *etpA*-negative genomes in Genbank (S3 Table) [51] demonstrated >96.6% sequence identity and retention of the catalytic triad.

**Fig 6:**
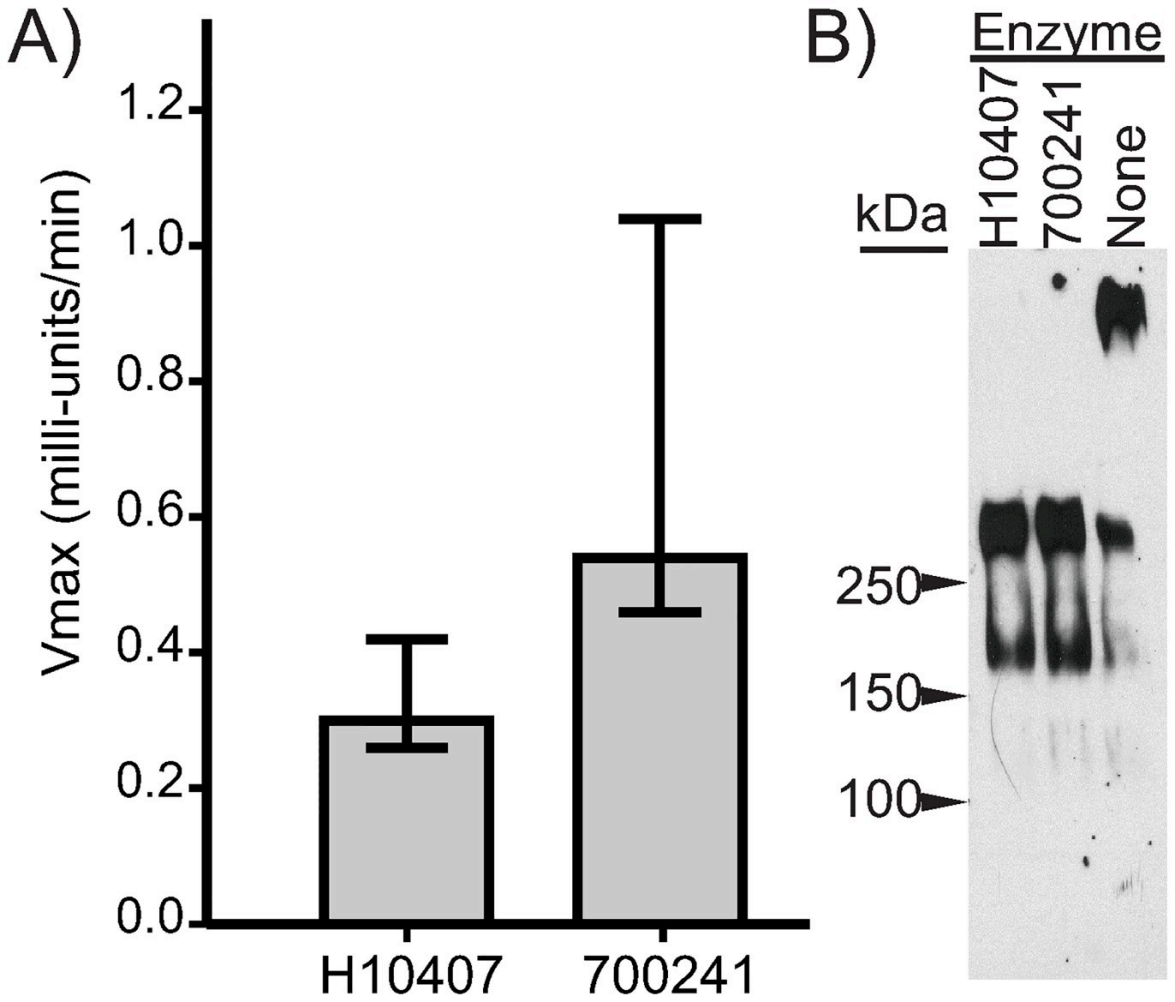
Mucinase activity of EatA is retained. (A) Purified recombinant EatA degrades the synthetic protein Suc-Ala-Ala-Pro-Leu-p-anilimide in a colorimetric assay measuring the rate of release of p-anilimide. 2 independent experiments with 3 replicates total, medians with 95% CI are shown, (p = 0.1, Mann-Whitney U). (B) Degradation of MUC2 by recombinant EatA from H10407 and strain 700241. Purified EatA and MUC2 from LS174T cells (~540 kDa) were incubated together with mucin degradation (~300 kDa) assessed by western blotting, 2 independent experiments performed.

The most divergent *eatA* sequence was cloned and recombinant passenger domain of EatA (rEatAp) was then purified to assess proteolytic activity. We first determined the proteolytic activity of EatA using the synthetic peptide Suc-Ala-Ala-Pro-Leu (AAPL) coupled to p-nitroanilide which when enzymatically cleaved produces the readily detectable yellow indicator, 4-nitroaniline [25]. We found that similar to EatA from H10407, rEatAp from ETEC strain 700241 cleaved AAPL, (Fig 6A). Likewise, rEatAp derived from H10407 and 700241 degraded MUC2 (Fig 6B), providing further evidence that the minor degrees of sequence divergence observed within the predicted EatA peptide sequence did not affect functional activity of the enzyme.

## Discussion

The recognized diversity of canonical target molecules and the potential complexity inherent in formulating broadly protective ETEC vaccines has driven the pursuit of novel strategies [52, 53]. Emerging pathogenesis studies suggest that additional surface-expressed antigens could complement ETEC canonical CF-centered approaches. Genetic loci encoding two secreted virulence proteins not currently targeted in ETEC vaccines, EatA and EtpA, were initially discovered in ETEC H10407 on the same plasmid that encodes CFA/I [54]. Studies thus far have demonstrated that these antigens contribute to ETEC virulence, are highly immunogenic during natural and experimental infections, and afford protection against infection in an animal model [28, 31–33]. These promising initial results led to current efforts to ensure that these antigens are sufficiently conserved across isolates to warrant further investigation of their utility as vaccine antigens. As molecular conservation remains an essential benchmark in evaluating candidate antigens, the present studies were performed to provide more accurate estimates of the global distribution and molecular sequence conservation of EtpA and EatA. To avoid limitations of earlier studies derived from one geographic location or biased collections where the intent is to maximize diversity [33, 55–57], we interrogated well-validated ETEC isolates of known provenance from symptomatic illness collected over a broad geographic distribution. Our findings affirm the conclusions from the prior studies and expand the knowledge base to more diverse geographies.

The studies reported here suggest that both EtpA and EatA are among the most highly conserved ETEC pathovar-specific antigens described to date. Moreover, by sequencing a geographically and phenotypically diverse subset of ETEC isolates we found that both antigens exhibit substantial molecular sequence conservation and retain core virulence functions across a range of isolates. Ongoing work seeks to identify conserved and protective epitopes for rational vaccine design. Collectively, these features could simplify rational vaccine design.

No ETEC pathovar-specific protein, including either the LT or ST toxins that define ETEC, is universally conserved in every strain. Because ST-producing strains appear to predominate among ETEC associated with symptomatic illness, recent studies have focused on the prevalence of canonical vaccine target antigens among ST+ETEC. Similar to canonical CF targets, we also find that *eatA* and *etpA* are more frequently associated with ST-producing strains relative to LT-only isolates. Both loci were originally identified on the p948 plasmid in ETEC H10407 which encodes *eatA*, *etpA*, and *sta2* (STh), while the p666 plasmid encodes *sta1* (STp) and genes for LT (*eltA* and *eltB*). Although it is possible that the *eatA* and *etpBAC* loci are similarly linked on plasmids in other strains, the resolution of present sequencing data is not sufficient to support the contention that these loci are inextricably linked to those encoding STh.

It is important to examine the findings of the present study relative to other potential target molecules. Data from recent immunoproteome studies have demonstrated that ETEC infection induces mucosal antibody responses to EatA, EtpA, as well as canonical ETEC vaccine antigens [32]. Notably, these studies also detected immune responses to highly conserved, chromosomally encoded proteins, such as flagellin [58], making them potentially attractive candidate antigens that could protect against multiple *E*. *coli* pathovars in addition to ETEC. Whether vaccination with these core antigens, which are shared with the small population of commensal *E*. *coli*, would adversely impact the human intestinal microbiota is currently unknown [59]. Likewise, it is not known whether the immune response to these conserved proteins that follows natural infections with ETEC could contribute to non-diarrheal sequelae linked to these infections. At present, selectively targeting a compilation of pathovar-specific antigens could offer a rational approach to a vaccine that affords broad-based coverage.

The ideal strategy for incorporation of these antigens into the development of a highly efficacious and broadly protective vaccine will need to be determined. However, EtpA and EatA could be adopted in a multivalent approach that targets complementary virulence characteristics and extends antigenic valency. Similarity in virulence strategies between ETEC and *Bordetella pertussis* [60] may offer an important template for rational vaccine development. Both pathogens produce toxins which induce cyclic nucleotides (pertussis toxin vs LT/ST), utilize two-partner secretion hemagglutinin exoprotein adhesin molecules (filamentous hemagglutinin [FHA] vs EtpA), fimbrial adhesins (pertussis fimbriae vs CFs), and surface expressed or secreted autotransporter molecules important for virulence (pertactin vs EatA). Present acellular pertussis vaccines that incorporate toxoids, fimbriae, pertactin, and FHA [61] could therefore provide a valuable paradigm for the rational design of ETEC vaccines that combine novel antigens highlighted here with emerging LT and ST toxoids, and CFs selected to achieve broad coverage against a diverse population of ETEC.

The broad representation of EatA and EtpA in a diverse and geographically distributed population of ETEC isolated from symptomatic cases of diarrheal illness would appear to support recent *in vitro*, and animal studies, as well as controlled human infection model data suggesting that these antigens play an important role in the molecular pathogenesis of disease. These findings combined with the established immunogenicity of these proteins should encourage further examination of their role as protective antigens.

## Supporting information

S1 Supporting methods (PDF)

S1 Table Primers used in this publication. (PDF)

S2 Table Database of isolates analyzed in this study. (XLSX)

S3 Table List of sequenced isolates and their GenBank IDs. (XLSX)

S4 Table Bacterial strains and plasmids in this publication. (PDF)

S5 Table Antigen distribution by study origin. (PDF)

S1 Fig Flow diagram depicting exclusion of samples. Isolates not meeting PCR criteria for inclusion are defined as having non-specific amplification of toxins despite a minimum of 3 assays. Isolates were determined not to be ETEC if they lacked detectable toxin in the original report and on repeat testing at Washington University. All of these prior isolates were reported to express ETEC specific CFs without toxins. The remaining 51 strains lacked detectable toxin at Washington University, suggesting loss of plasmid. UoB is University of Buffalo (Colombian isolates), MNDOH is Minnesota Department of Health.
(PDF)

S2 Fig Phylogenomic analysis of EtpA-expressing ETEC strains sequenced in this study compared with a collection of previously sequenced ETEC reference genomes and diverse *E*. *coli* and *Shigella* genomes. A maximum likelihood phylogeny was generated from 217,248 SNPs relative to the genome of *E*. *coli* IAI39 as a reference. The newly sequenced EtpA-expressing ETEC genomes are indicated in red. *E*. *coli* phylogroups (A, B1, B2, D, E, and F) are indicated on the interior of the phylogeny, while ETEC phylogenomic lineages (L1 through L21) that contain an EtpA-expressing isolate are indicated on the exterior of the phylogeny. The presence of *eatA* and *etpA* is indicated by blue and orange squares (see inset legend). Bootstrap values ≥80 are indicated by a gray circle over each respective node.
(PDF)

S3 Fig EatA sequence alignment. Sequence alignment for EatA expressing isolates (S3 Table) included in this study. Arrows highlight the conserved catalytic triad H124, D164 (page 2), and S267 (page 3). The EatA passenger domain is annotated below the sequence on pages 1 and 10.
(PDF)
