# Supplementary material for: Conservation and global distribution of non-canonical antigens in Enterotoxigenic Escherichia coli
Source: PLoS Negl Trop Dis. 2019 Nov 22;13(11):e0007825. doi: 10.1371/journal.pntd.0007825 (PMC6897418; doi:10.1371/journal.pntd.0007825)
Supplement: S1 Supporting methods — (PDF) [file pntd.0007825.s001.pdf]

Supplemental Methods

Antigen detection by PCR and DNA extraction.

3 ml Luria Broth cultures were inoculated in a laminar flow hood and two separate negative control cultures were inoculated to validate sterile technique, a blank culture from the stock bottle of LB and a blank culture used to cool the wire loop prior to sampling from the frozen stock vial. Cultures were incubated overnight at 37°C. Total nucleic acids were extracted from 1.2 ml sample of these cultures using the Wizard Genomic DNA Purification Kit (Promega catalog #A1120). Total nucleic acids were resuspended in a total volume of 200µl water. Repeat testing on toxin discordant isolates or ambiguous results was performed using DNA that was extracted by standard phenol/chloroform extraction where bacteria harvested from 1.5 ml of culture was centrifuged at max speed for 5 minutes and resuspended in 500 µl of 50mM Tris, pH 8, with 2% sodium dodecyl sulfate and 200 µg of proteinase K, then incubated at 50°C for 30 minutes. 500 µl of phenol was added and the sample was gently mixed until homogenous followed by centrifugation at 4°C for 10 minutes at 9,400 x *g*. The top aqueous layer was removed and 500 µl of chloroform was added, gently mixed, and centrifuged as above. The top layer was again removed, mixed with 2 volumes of ice cold ethanol with gentle mixing and centrifugation. The supernatant was discarded and the pellet was washed once in 70% ethanol, allowed to air dry, and dissolved in 100 µl of 10 mM Tris, 1 mM EDTA, and 10 µg/ml RNAse A. PCR assays were performed using the GoTaq Green Master Mix (Promega M7122). Primers (Table S3) were used at a final concentration of 500 nM along with 1 µl of a 100-fold dilution of gDNA in a final reaction volume of 20 µl. For *eatA* and *etpA*, gDNA was denatured for 5 minutes at 95°C followed by 30 cycles of amplification using 95°C for 30 seconds, annealing at 52°C for 30 seconds, and extension at 72°C for 2 minutes. The toxin multiplex assay (eltB and sta1) and sta2 reactions were denatured for 5 minutes at 95°C followed by 32 cycles of

amplification using 94°C for 15 seconds, annealing at 55°C for 15 seconds, and extension at 72°C for 30 seconds. Amplicons were visualized on a 0.8% agarose gel with ethidium bromide.

Immunoblotting

Supernatants from overnight cultures described above were collected and proteins precipitated in 2.7M trichloroacetic acid for 30 min at 4°C. Proteins were pelleted at 18,400 x *g* for 5 minutes and washed twice in 200 µl of 4°C acetone then dried by heating for 5-10 minutes at 95°C. Pellets were dissolved in 25 µl of Laemmli buffer with 5% beta-mercaptoethanol and denatured by heating for 5 minutes at 95°C. Proteins were separated using 10% SDS-PAGE with H10407 (EatA and EtpA positive strain) protein precipitate as a positive control. Proteins were transferred to a nitrocellulose membrane using a Trans-Blot system (Bio-Rad) and membranes were blocked for 60 minutes in 5% milk containing PBS-Tween (0.05%) at room temperature. Affinity purified rabbit anti-EatA (1) or rabbit anti-EtpA antibodies (2) were diluted blocking solution and incubated at 4°C overnight. Membranes were washed 3 times in PBS-T and primary antibodies detected using HRP-conjugated anti-rabbit IgG secondary antibody (1:5,000 dilution, Invitrogen #A16110) diluted in blocking solution for 1 hour at room temperature. Membranes were then developed using a luminol based ECL western blotting substrate (Bio-Rad, #ABIN412579).

Strains and plasmids

etpBA from strains 500662 and 201600538.1 were amplified using primers 031505.1 and 110705.2 followed by gel purification and transformation into pCR-Blunt II-TOPO vector (Thermo Fisher) in Top10 cells. Purified plasmid was digested with HindIII and XhoI (New England Biolabs) and ligated into the expression vector pBAD-myc-HisA (Invitrogen) with transformation into Top10 cells. Purified plasmid was transformed into Top10 cells (jf1477) containing the expression vector for EtpC from H10407 (pACYC184) generating strains jf4927 (EtpA from 500662) and jf4894 (EtpA from 201600538.1). *eatA* from strain 700241 was amplified by primers (jf53018.1 and jf53018.2) and gel purified while pBAD-myc-HisA was

linearized with XhoI and NcoI. These fragments were assembled using the Gibson Assembly Cloning Kit (NEB: catalog #E5510S) and transformed into NEB 5-alpha competent cells. Purified expression plasmid was then transformed into jf2827 FliC mutant cells for recombinant protein expression and purification as previously described with the omission of ammonium sulfate precipitation.

Phylogenomic analysis

The 46 *etpA* containing ETEC genomes sequenced in this study were analyzed by phylogenomic analysis compared to a reference collection of 61 previously sequenced ETEC genomes and 32 diverse *E. coli* and *Shigella* genomes as previously described (3). The *In Silico* Genotyper (ISG) was used to identify single nucleotide polymorphisms (SNPs) in each genome relative to the reference genome *E. coli* IAI39 (GenBank accession number NC_011750.1) as previously described (3, 4). There were 217,248 SNP sites in each genome that were concatenated and used to construct a phylogeny. A maximum-likelihood phylogeny was inferred using RAxML v7.2.8 (5), with the GTR model of nucleotide substitution, the GAMMA model of rate heterogeneity, and 100 bootstrap replicates. The phylogeny was then mid-point rooted and decorated using the interactive tree of life (iTOL) v.4 software (6).

Functional Assays.

HT-29 cells and the CRISPR α1-3-*N*-acetylgalactosaminyltransferase deletion line, IE6, were grown in McCoy's 5a media (Gibco) supplemented with 10% fetal bovine serum for 72 hours on 8-well Millicell EZ Slides (Millipore Sigma PEZGS0816) at 37°C with 5% CO_2 as before (7). Cells were fixed in 4% paraformaldehyde for 10 minutes, washed in PBS, and incubated with 50 µg of EtpA. Cells were then washed in PBS and blocked in 2% BSA for 30 minutes. After washing x3 in PBS, affinity purified rabbit anti-EtpA IgG antibodies (1:50 dilution) or mouse anti-Blood Group A IgM Ab (1:40, Santa Cruz Biotechnology Inc., sc-69951) were used to detect EtpA and blood group sugars to verify blood group expression. One hour later, cells were washed and counterstained with 1:200 dilution of Alexaflour 488 anti-mouse IgM or Alexflour

597 anti-rabbit IgG antibodies (Invitrogen) with 1:2,000 dilution of CellMask red membrane stain (Thermo Fisher) and 1:6,000 dilution of DAPI. Cells and staining were preserved using Prolong Gold Anti-Fade reagent (Invitrogen). A minimum of 10 randomly obtained images were processed using Volocity software (v6.3) to determine the total fluorescence intensity of the anti-EtpA signal normalized to the total DAPI signal as a surrogate for cell number.

EatA activity was assayed with purified recombinant EatA (30 µg) in 100 mM MOPS, 200 mM NaCl pH 7.3 buffer at 37°C and the reaction initiated by the addition of 20 µl of 10 mM N-Succinyl-Ala-Ala-Pro-Leu p-nitroanilide (Sigma, S8511) in a final reaction volume of 200 µl. The release of 4-nitroanilline product was followed for 3 hours at 405 nm using an Eon microplate reader (BioTek, VT) as previously described(1). Data was analyzed using Gen5 version 2.0 software (Biotek). Mucin was purified from culture supernatants of LS174T cells (ATCC CL-188) grown in Eagle's media supplemented with 10% FBS and grown at 37°C with 5% CO_2. Size exclusion chromatography was performed on concentrated supernatant with MUC2 fractions confirmed by Western Blotting as previously described (8). 20 µg of purified mucin was incubated with 6 ug of recombinant EatA in 60 µl reaction volumes and incubated at 37°C for 20 minutes. Samples were run on 3-8% NuPage Gradient gels (Thermo Fisher, EA03752). Blots were developed using anti-MUC2 rabbit polyclonal (IgG) antibody (1:2,000 dilution, Santa Cruz H-300 sc-15334) at 4°C overnight followed by detection with HRP-conjugated anti-rabbit IgG secondary antibody as above.

Supporting References:

1. Patel SK, Dotson J, Allen KP, Fleckenstein JM. Identification and molecular characterization of EatA, an autotransporter protein of enterotoxigenic Escherichia coli. Infect Immun. 2004;72(3):1786-94.
2. Fleckenstein JM, Roy K, Fischer JF, Burkitt M. Identification of a two-partner secretion locus of enterotoxigenic Escherichia coli. Infect Immun. 2006;74(4):2245-58.
3. Hazen TH, Nagaraj S, Sen S, Permala-Booth J, Del Canto F, Vidal R, et al. Genome and Functional Characterization of Colonization Factor Antigen I- and CS6-Encoding Heat-Stable Enterotoxin-Only Enterotoxigenic Escherichia coli Reveals Lineage and Geographic Variation. mSystems. 2019;4(1).

4. Sahl JW, Beckstrom-Sternberg SM, Babic-Sternberg J, Gillece JD, Hepp CM, Auerbach RK, et al. The In Silico Genotyper (ISG): an open-source pipeline to rapidly identify and annotate nucleotide variants for comparative genomics applications. bioRxiv. 2015:015578.
5. Stamatakis A. RAxML-VI-HPC: maximum likelihood-based phylogenetic analyses with thousands of taxa and mixed models. Bioinformatics. 2006;22(21):2688-90.
6. Letunic I, Bork P. Interactive Tree Of Life (iTOL) v4: recent updates and new developments. Nucleic Acids Res. 2019.
7. Kuhlmann FM, Santhanam S, Kumar P, Luo Q, Ciorba MA, Fleckenstein JM. Blood Group O-Dependent Cellular Responses to Cholera Toxin: Parallel Clinical and Epidemiological Links to Severe Cholera. Am J Trop Med Hyg. 2016;95(2):440-3.
8. Kumar P, Luo Q, Vickers TJ, Sheikh A, Lewis WG, Fleckenstein JM. EatA, an immunogenic protective antigen of enterotoxigenic Escherichia coli, degrades intestinal mucin. Infect Immun. 2014;82(2):500-8.
