# Supplementary material for: Conservation and global distribution of non-canonical antigens in Enterotoxigenic Escherichia coli
Source: PLoS Negl Trop Dis. 2019 Nov 22;13(11):e0007825. doi: 10.1371/journal.pntd.0007825 (PMC6897418; doi:10.1371/journal.pntd.0007825)
Supplement: S1 Table — (PDF) [file pntd.0007825.s002.pdf]

S1 Table. Primers used in this publication

Gene	Product size (bp)	Sequence (5'>3')	Fleckenstein Lab ID
eatA (GenBank: AY163491.2)	1943	ATGTGCTTTGGCAGGTTAA	jf082213.1-F
		ATATCCAGTCAGCACCCACT	jf082213.2-R
etpA (GenBank: AY920525.2)	999	GGTTCAGGCAGTATCCAGAC	jf082213.3-F
		GGTGTAGCTGTCTGACCACA	jf082213.4-R
eltB (GenBank: CBJ04425.1)	273	ACGGCGTTACTATCCTCTC	jf092313.3-F
		TGGTCTCGGTCAGATATGTG	jf092313.4-R
sta1 (STp, GenBank: CBJ04435.1)	166	TCTTTCCCCTCTTTTAGTCAG	jf092313.5-F
		ACAGGCAGGATTACAACAAAG	jf092313.6-R
sta2 (STh, GenBank: CBJ04483.1)	64	TACAAGCAGGATTACAACAC	jf092313.7-F
		AGTGGTCCTGAAAGCATG	jf092313.8-R
etpBA (GenBank: AY920525.2)	6521	AATAATCTCGAGaATGGTGGTGAAATTCATG	jf031505.1-F
		AATAATAAGCTTTTGCCAGTACACCTCACT	jf110705.2-R
eatA (GenBank: AY163491.2)	4136	GGCTAACAGGAGGAATTAACCATGAATA AAGTGTTCTC TCTTAAG	jf053018.1-F
		TATGGTACCAGCTGCAGATCTCAGAAAT AATAACGGAAGTTAG	jf053018.2-R
