# Supplementary material for: Conservation and global distribution of non-canonical antigens in Enterotoxigenic Escherichia coli
Source: PLoS Negl Trop Dis. 2019 Nov 22;13(11):e0007825. doi: 10.1371/journal.pntd.0007825 (PMC6897418; doi:10.1371/journal.pntd.0007825)
Supplement: S4 Table — (PDF) [file pntd.0007825.s005.pdf]

S4 Table. Bacterial strains and plasmids in this publication

Strain or plasmid	Description	Reference
Strain		
5-alpha		New England Biolabs
Top10	F$^-$ *mcrA* Δ(*mrr-hsdRMS-mcrBC*) Φ80*lacZ*ΔM15 Δ*lacX74* *recA1 araD139* Δ(*ara leu*)*7697 galU galK rpsL* (Str^r)	Invitrogen
jf1477	Top10 (pJL030, pJL017)	(1)
jf4927	Top10 cells (pJL030, pBad-myc-HisA_3954)	This study
jf4894	Top10 cells (pJL030, pBad-myc-HisA_3645)	This study
jf2827	LMG194ΔfliC	(2)
jf5003	LMG194ΔfliC (pBAD-myc-HisA-3813EatA	This study
Plasmids		
pBAD-myc-HisA	Arabinose inducible expression system	Invitrogen
pJL030	*etpC* gene cloned into pACYC184; Cm^r	(3)
pJL017	*etpBA* gene cloned into pBAD-myc-HisA; Amp^r	(3)
pCR-Blunt II-TOPO	*ccdB* Kan^r Phleo^r cloning vector	Thermo Fisher
pBAD-myc-HisA_3954	*etpBA* gene from 500662 cloned into pBAD-myc-HisA; Amp^r	This study
pBAD-myc-HisA_3645	*etpBA* gene from 201600538.1 cloned into pBAD-myc-HisA; Amp^r	This study
pBAD-myc-HisA-3813EatA	*eatA* gene from 700241 cloned into pBAD-myc-HisA; Amp^r	This study

Table References:

1. Roy K, Hilliard GM, Hamilton DJ, Luo J, Ostmann MM, Fleckenstein JM. Enterotoxigenic Escherichia coli EtpA mediates adhesion between flagella and host cells. Nature. 2009;457(7229):594-8.

2. Roy K, Kansal R, Bartels SR, Hamilton DJ, Shaaban S, Fleckenstein JM. Adhesin degradation accelerates delivery of heat-labile toxin by enterotoxigenic Escherichia coli. J Biol Chem. 2011;286(34):29771-9.

3. Fleckenstein JM, Roy K. Purification of recombinant high molecular weight two-partner secretion proteins from Escherichia coli. Nat Protoc. 2009;4(7):1083-92.
