# Supplementary material for: Conservation and global distribution of non-canonical antigens in Enterotoxigenic Escherichia coli
Source: PLoS Negl Trop Dis. 2019 Nov 22;13(11):e0007825. doi: 10.1371/journal.pntd.0007825 (PMC6897418; doi:10.1371/journal.pntd.0007825)
Supplement: S5 Table — (PDF) [file pntd.0007825.s006.pdf]

S5 Table. Results of Antigen prevalence by study origin

Study or Collection	Total Isolates[a]	*eatA* or EatA			*etpA* or EtpA			Either *eatA*/EatA or *etpA*/EtpA		
		N[b]	%	95%CI	N	%	95%CI	N	%	95%CI
GEMS (1)	807	463	57.4	54.0-60.8	414	51.3	47.9-54.8	591	73.2	70.2-76.3
Minnesota Department of Health (2)	38	18	47.4	30.7-64.0	17	44.7	28.2-61.3	23	60.5	44.2-76.8
Bangladesh[c]	50	31	62.0	48.1-75.9	20	40.0	26.0-54.1	40	80.0	68.5-91.5
Chile (3)[d]	40	20	50.0	33.8-66.2	25	62.5	46.8-78.2	31	77.5	64.0-91.0
Mexico Travelers (4)	8	2	25.0	0-63.7	2	25.0	0-63.7	2	25.0	0-63.7
Fleckenstein Lab (5)[d]	180	105	58.3	51.1-65.6	101	56.1	48.8-63.4	138	76.7	70.4-82.9
Colombia (6)[d,e]	34	20	58.8	41.4-76.3	16	47.1	29.4-64.7	22	64.7	47.8-81.6

[a]Two isolates in this database came from outside sources not associated with any study and not included in this table (see S2 Table, ThroopD (7), and TW10598 (8))

[b]Number of positive isolates

[c]Samples obtained from routine culture surveillance

[d]Antigen prevalence in these isolates was previously reported

[e]Immunoblotting of these isolates was added in this report

Table References:

1. Kotloff KL, Nataro JP, Blackwelder WC, Nasrin D, Farag TH, Panchalingam S, et al. Burden and aetiology of diarrhoeal disease in infants and young children in developing countries (the Global Enteric Multicenter Study, GEMS): a prospective, case-control study. Lancet. 2013;382(9888):209-22.
2. Medus C, Besser JM, Juni BA, Koziol B, Lappi V, Smith KE, et al. Long-Term Sentinel Surveillance for Enterotoxigenic Escherichia coli and Non-O157 Shiga Toxin-Producing E. coli in Minnesota. Open Forum Infect Dis. 2016;3(1):ofw003.
3. Montero D, Vidal M, Pardo M, Torres A, Kruger E, Farfan M, et al. Characterization of enterotoxigenic Escherichia coli strains isolated from the massive multi-pathogen gastroenteritis outbreak in the Antofagasta region following the Chilean earthquake, 2010. Infect Genet Evol. 2017;52:26-9.
4. Mohamed JA, DuPont HL, Flores J, Palur H, Nair P, Jiang ZD, et al. Single nucleotide polymorphisms in the promoter of the gene encoding the lipopolysaccharide receptor CD14 are associated with bacterial diarrhea in US and Canadian travelers to Mexico. Clin Infect Dis. 2011;52(11):1332-41.
5. Luo Q, Qadri F, Kansal R, Rasko DA, Sheikh A, Fleckenstein JM. Conservation and immunogenicity of novel antigens in diverse isolates of enterotoxigenic Escherichia coli. PLoS Negl Trop Dis. 2015;9(1):e0003446.
6. Gomez-Duarte OG, Romero-Herazo YC, Paez-Canro CZ, Eslava-Schmalbach JH, Arzuza O. Enterotoxigenic Escherichia coli associated with childhood diarrhoea in Colombia, South America. J Infect Dev Ctries. 2013;7(5):372-81.
7. Finkelstein RA, Vasil ML, Jones JR, Anderson RA, Barnard T. Clinical cholera caused by enterotoxigenic Escherichia coli. J Clin Microbiol. 1976;3(3):382-4.
8. Steinsland H, Valentiner-Branth P, Perch M, Dias F, Fischer TK, Aaby P, et al. Enterotoxigenic Escherichia coli infections and diarrhea in a cohort of young children in Guinea-Bissau. J Infect Dis. 2002;186(12):1740-7.
