# Supplementary figures and images for: Conservation and global distribution of non-canonical antigens in Enterotoxigenic Escherichia coli
Source: PLoS Negl Trop Dis. 2019 Nov 22;13(11):e0007825. doi: 10.1371/journal.pntd.0007825 (PMC6897418; doi:10.1371/journal.pntd.0007825)

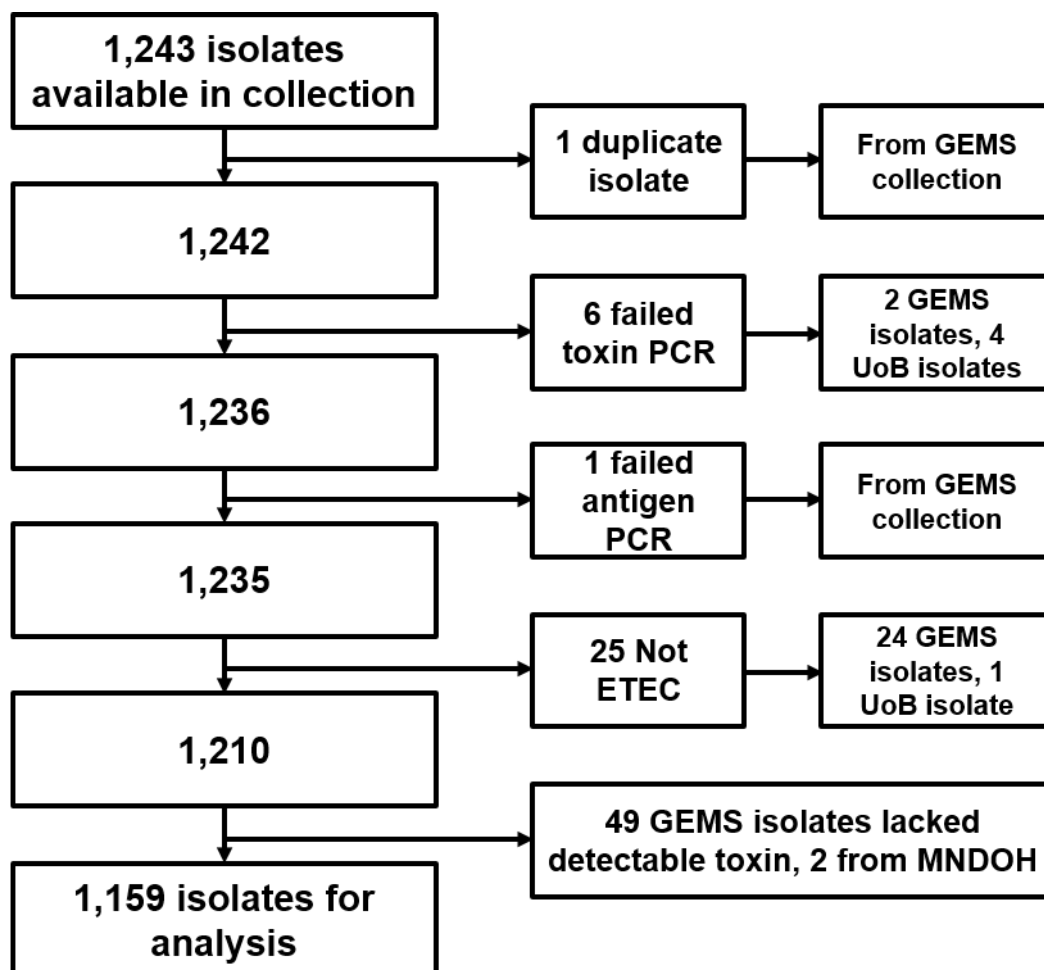

Supplement: S1 Fig — Isolates not meeting PCR criteria for inclusion are defined as having non-specific amplification of toxins despite a minimum of 3 assays. Isolates were determined not to be ETEC if they lacked detectable toxin in the original report and on repeat testing at Washington University. All of these prior isolates were reported to express ETEC specific CFs without toxins. The remaining 51 strains lacked detectable toxin at Washington University, suggesting loss of plasmid. UoB is University of Buffalo (Colombian isolates), MNDOH is Minnesota Department of Health. (PDF) [file pntd.0007825.s007.pdf]

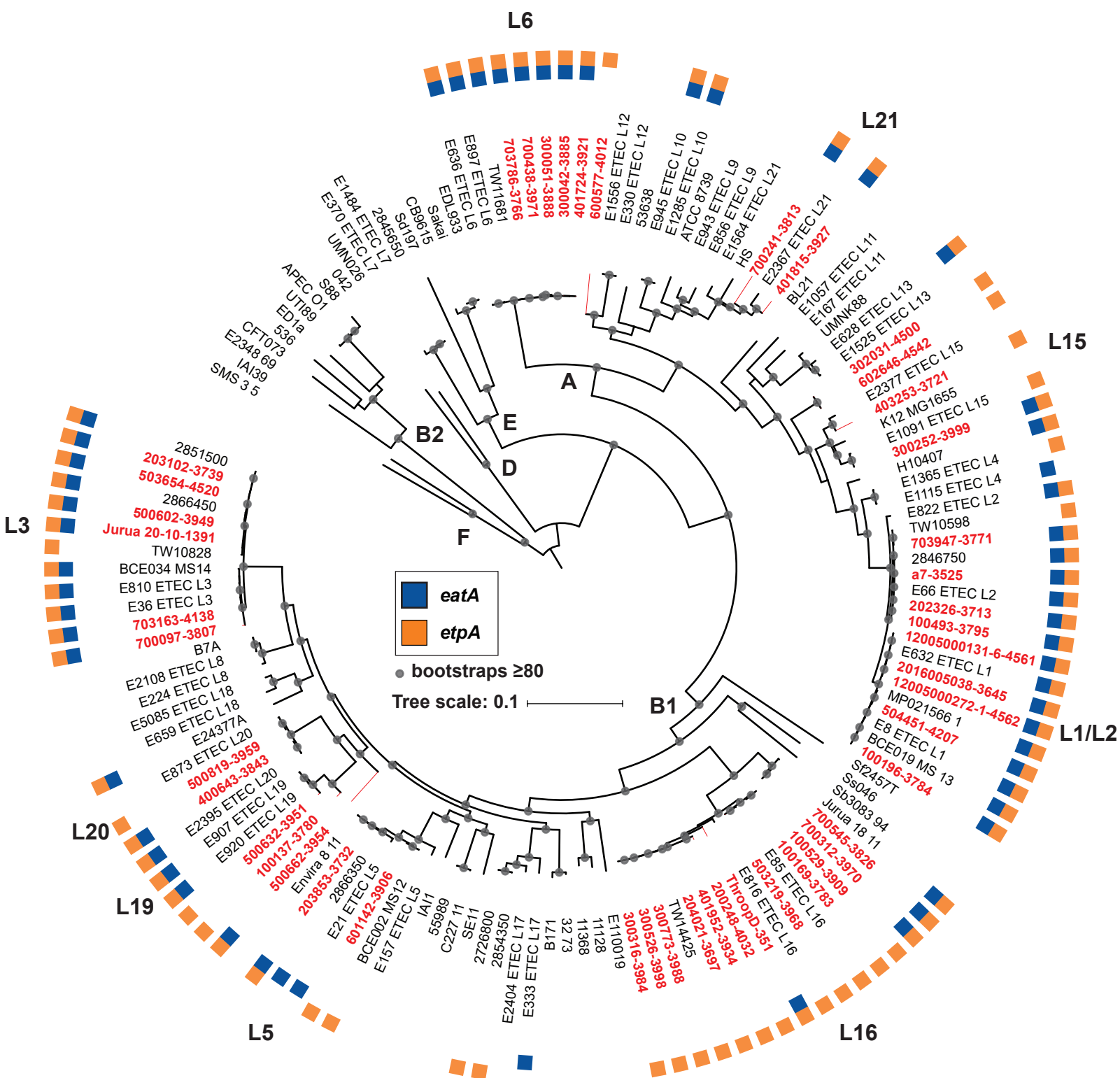

Supplement: S2 Fig — A maximum likelihood phylogeny was generated from 217,248 SNPs relative to the genome of E. coli IAI39 as a reference. The newly sequenced EtpA-expressing ETEC genomes are indicated in red. E. coli phylogroups (A, B1, B2, D, E, and F) are indicated on the interior of the phylogeny, while ETEC phylogenomic lineages (L1 through L21) that contain an EtpA-expressing isolate are indicated on the exterior of the phylogeny. The presence of eatA and etpA is indicated by blue and orange squares (see inset legend). Bootstrap values ≥80 are indicated by a gray circle over each respective node. (PDF) [file pntd.0007825.s008.pdf]

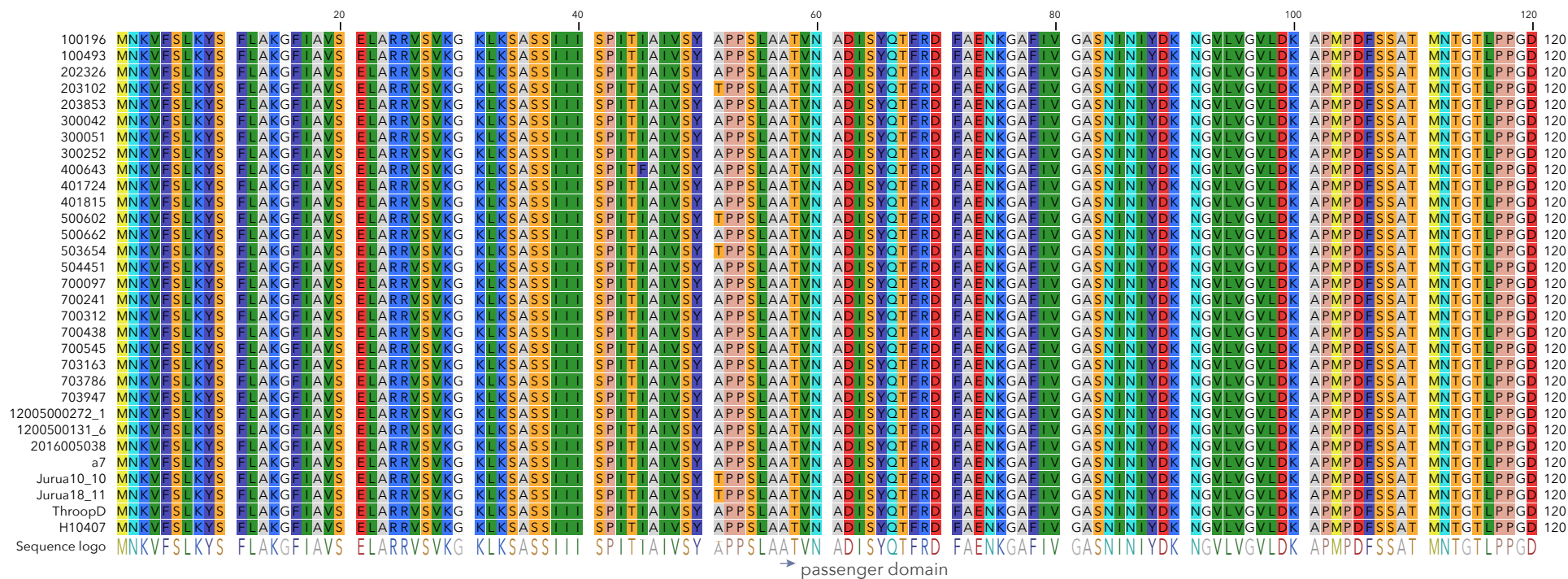

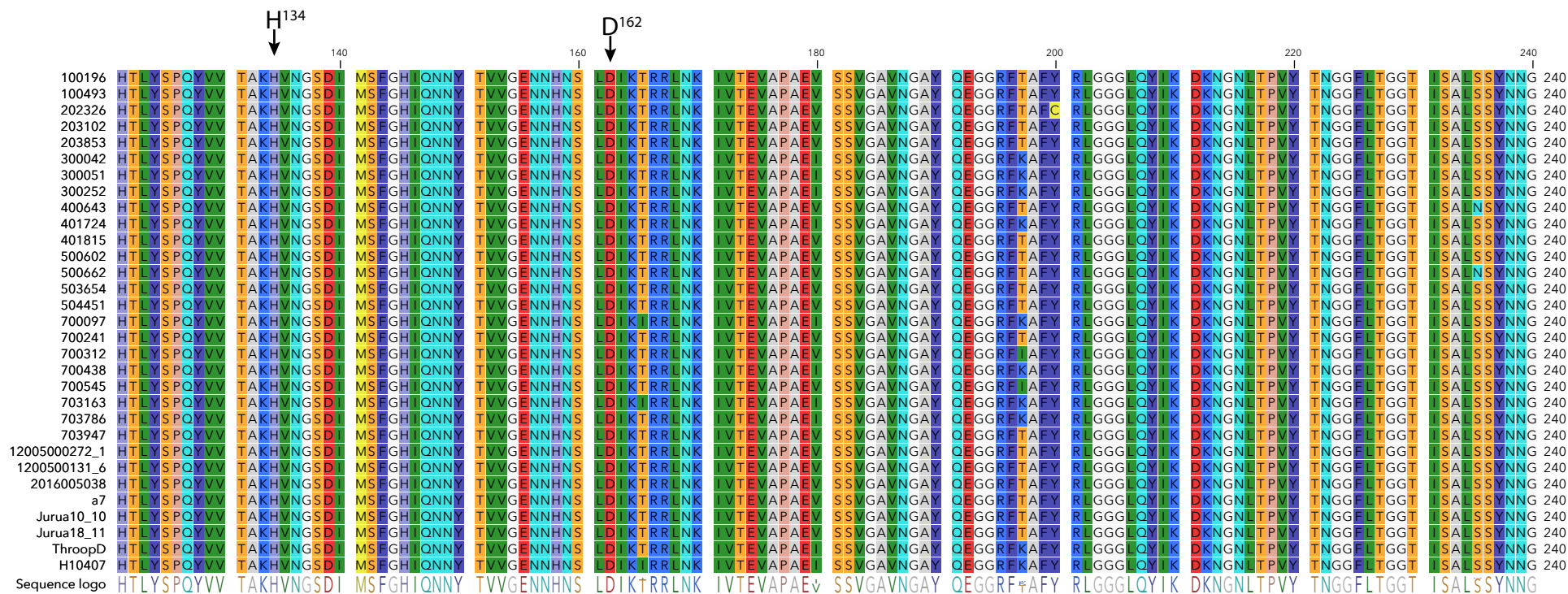

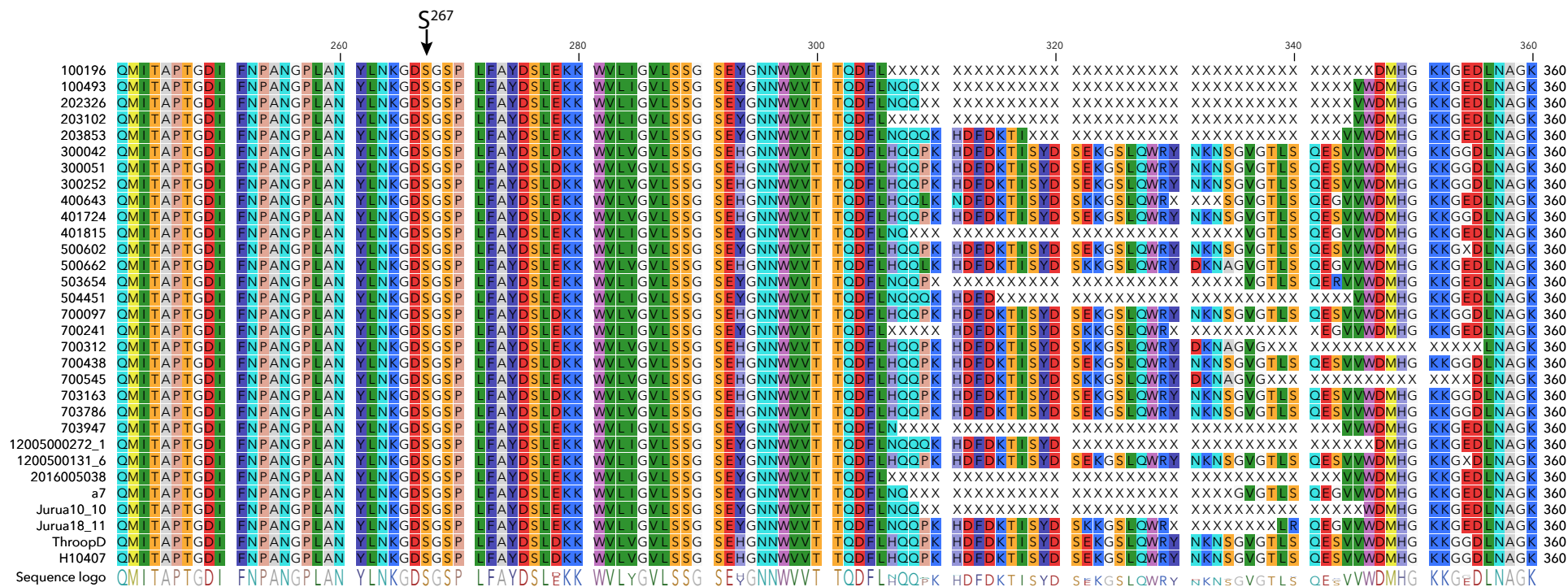

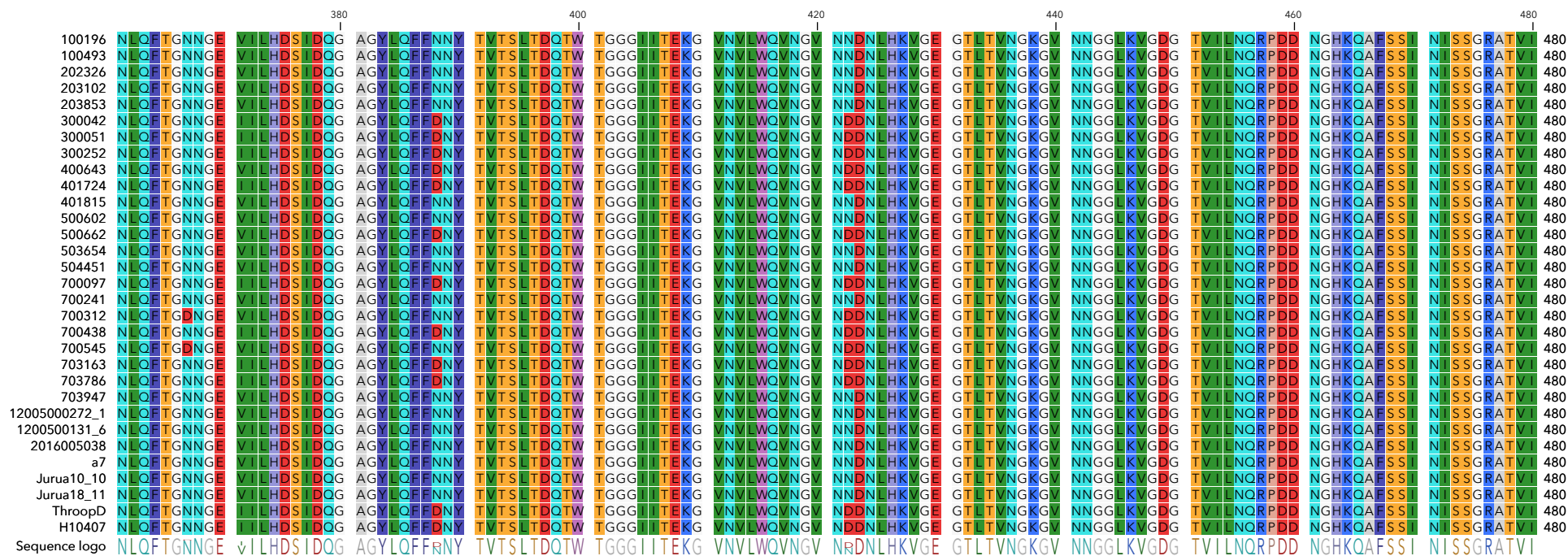

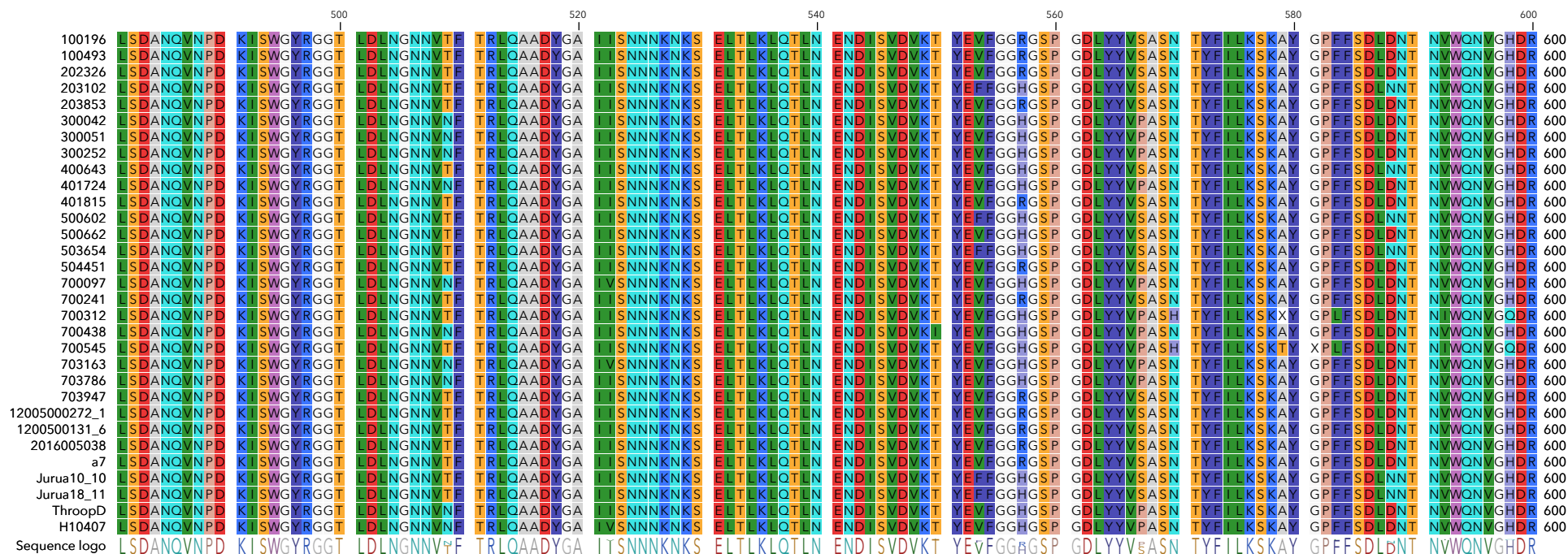

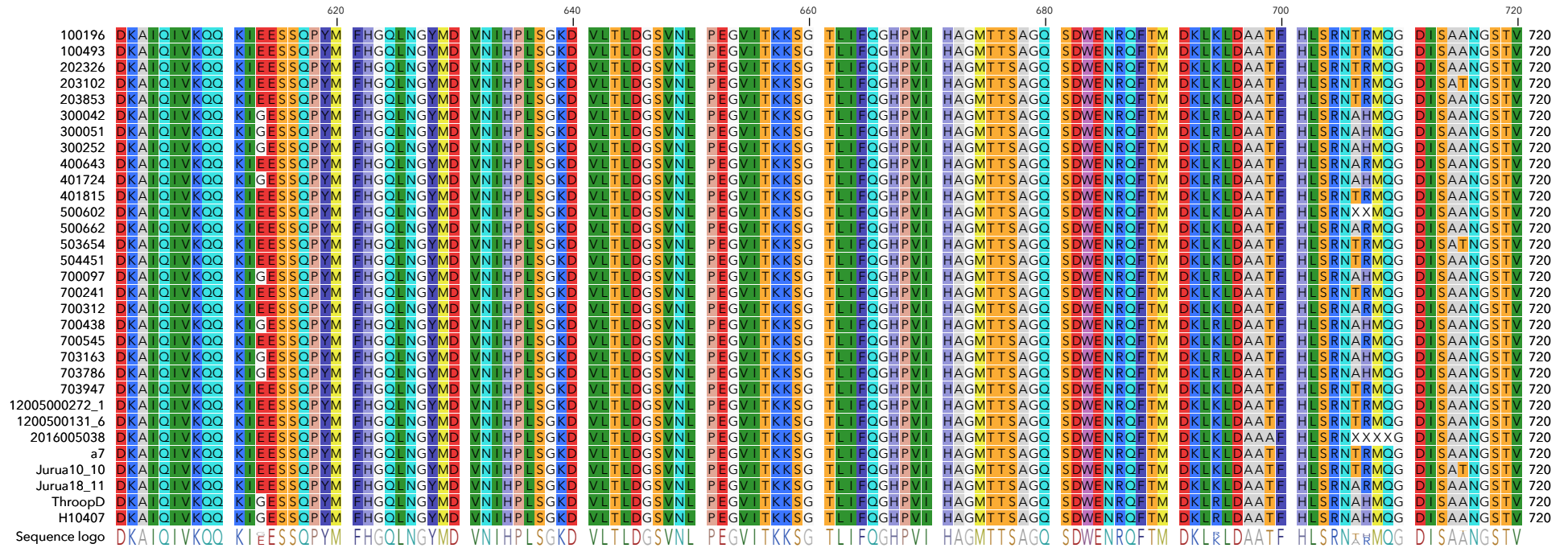

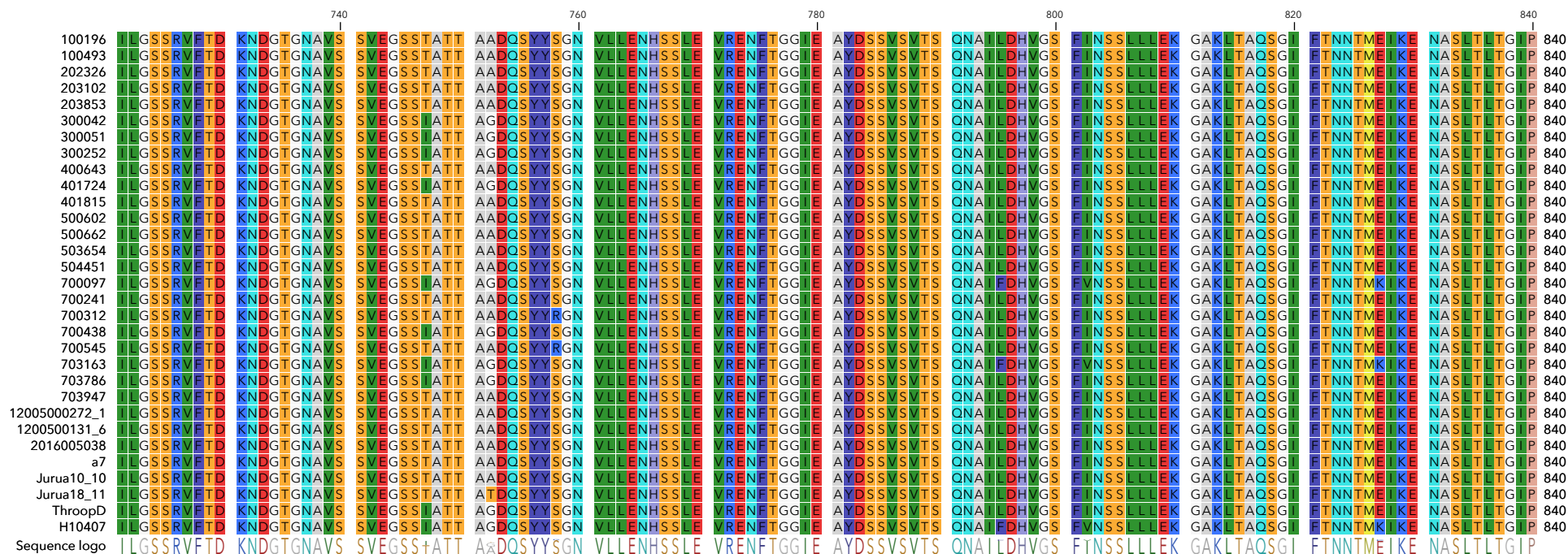

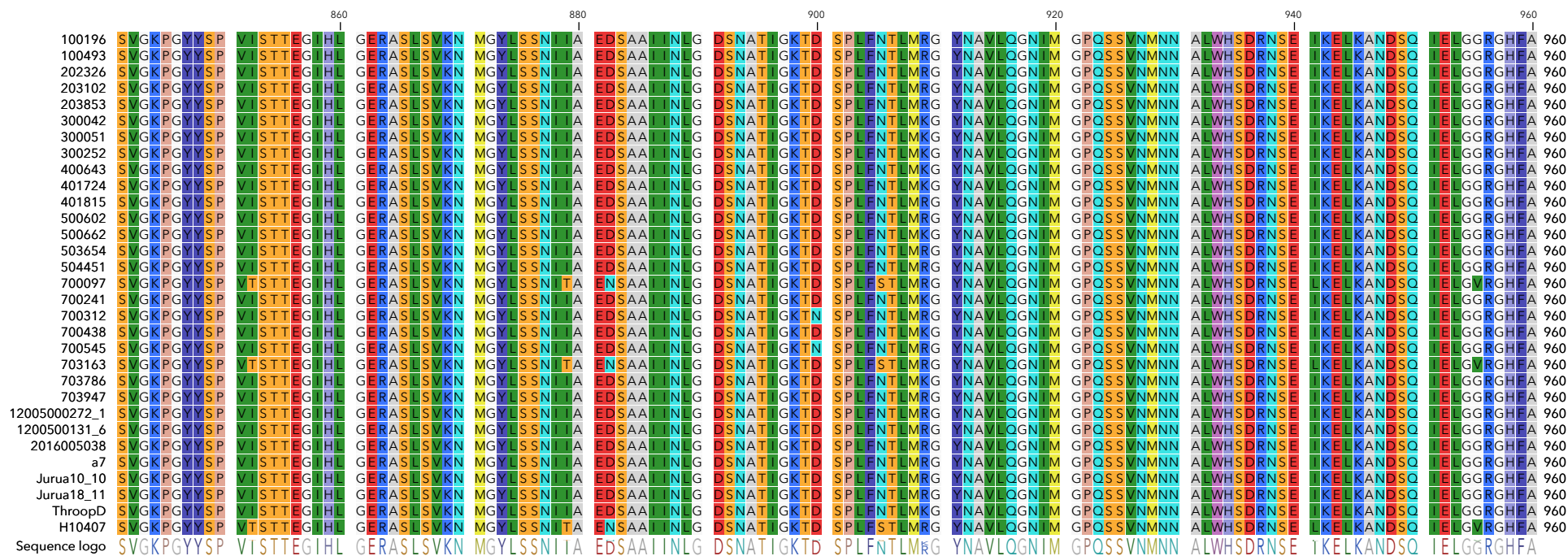

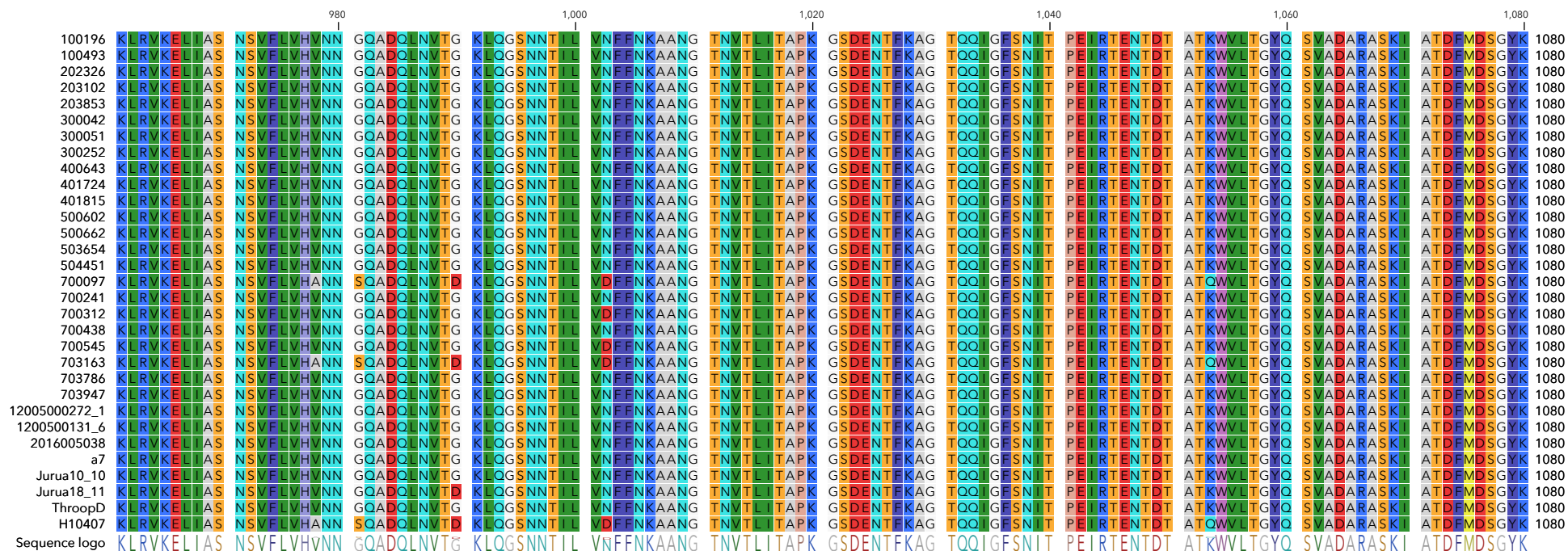

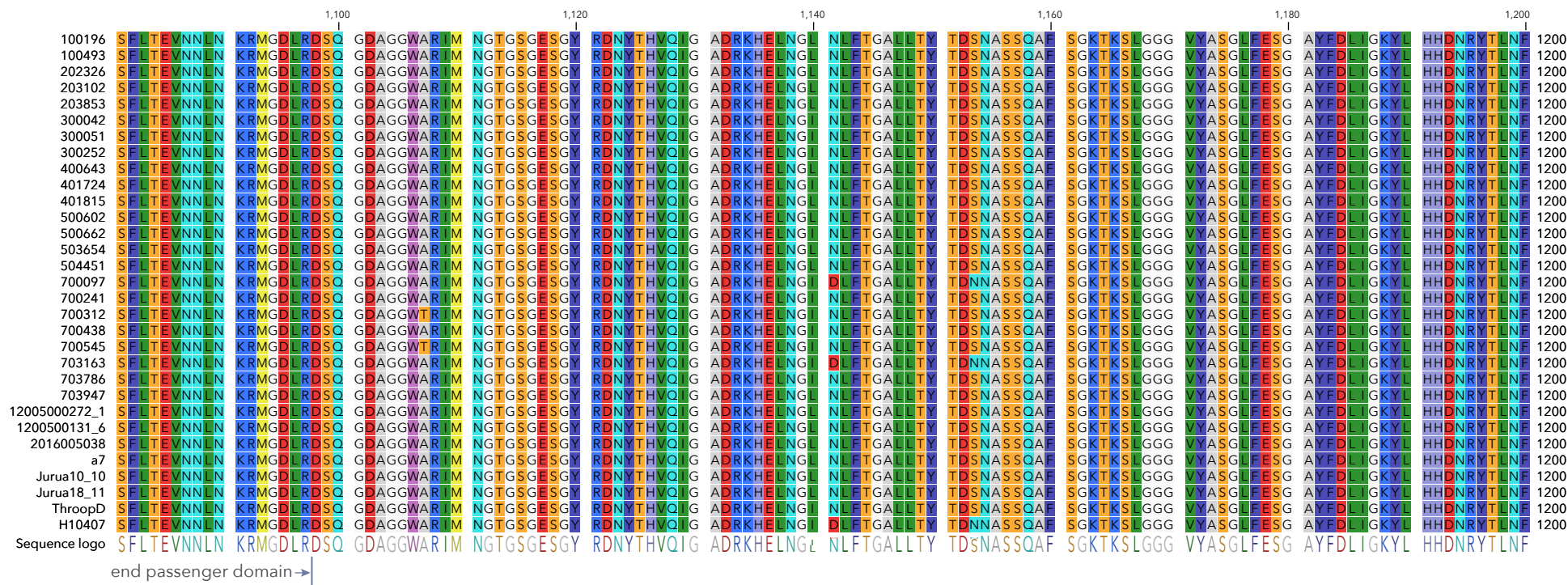



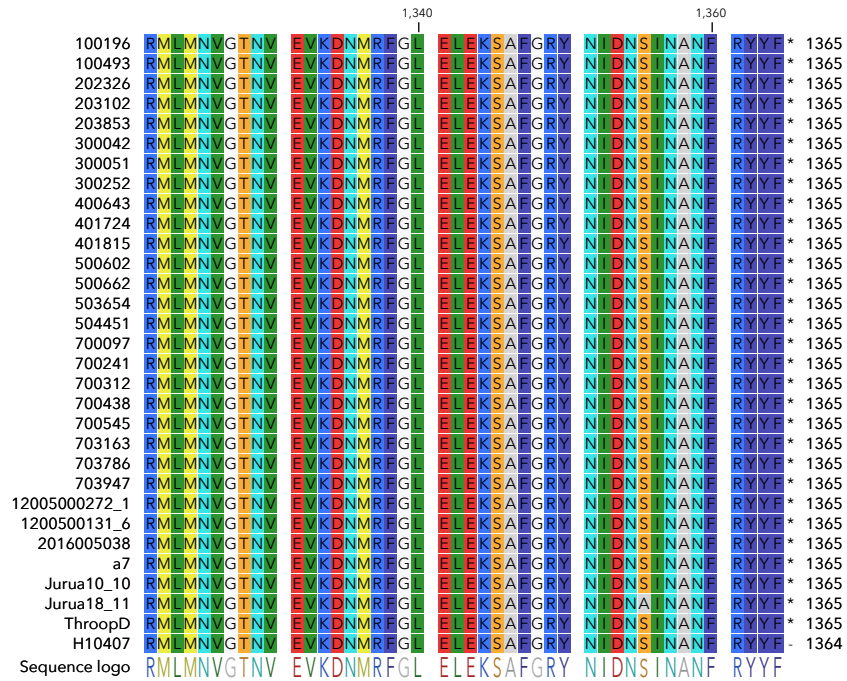

Supplement: S3 Fig — Sequence alignment for EatA expressing isolates (S3 Table) included in this study. Arrows highlight the conserved catalytic triad H124, D164 (page 2), and S267 (page 3). The EatA passenger domain is annotated below the sequence on pages 1 and 10. (PDF) [file pntd.0007825.s009.pdf]
